# Supplementary material for: Peptide derived from SLAMF1 prevents TLR4-mediated inflammation in vitro and in vivo
Source: Life Sci Alliance. 2023 Oct 3;6(12):e202302164. doi: 10.26508/lsa.202302164 (PMC10547912; doi:10.26508/lsa.202302164)

Source file for Figure 1

Peptide derived from SLAMF1 prevents TLR4-mediated inflammation *in vitro* and *in vivo*

Figure 1A, first panel, uncropped image for WCLs, ECF-tagged peptides

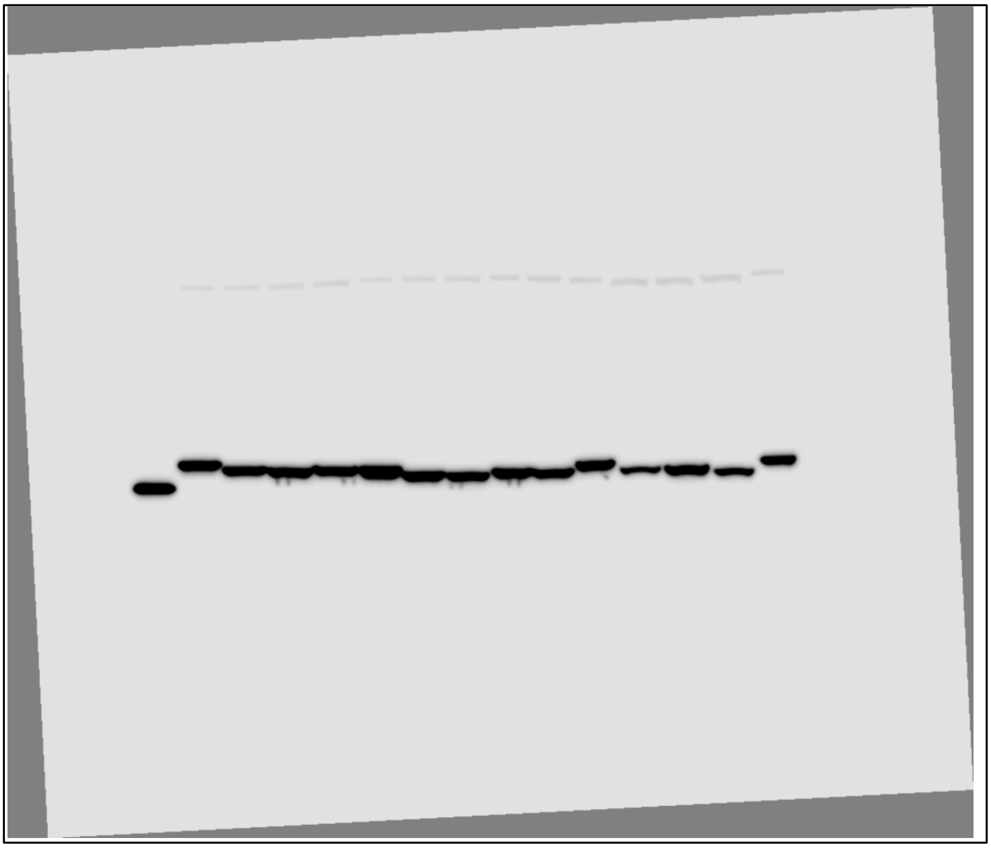

Figure 1A, second panel, uncropped image for WCLs, SLAMF1 (testing several lysates with SLAMF1 overexpression, first one used for the IPs represented on the Fig. 1A)

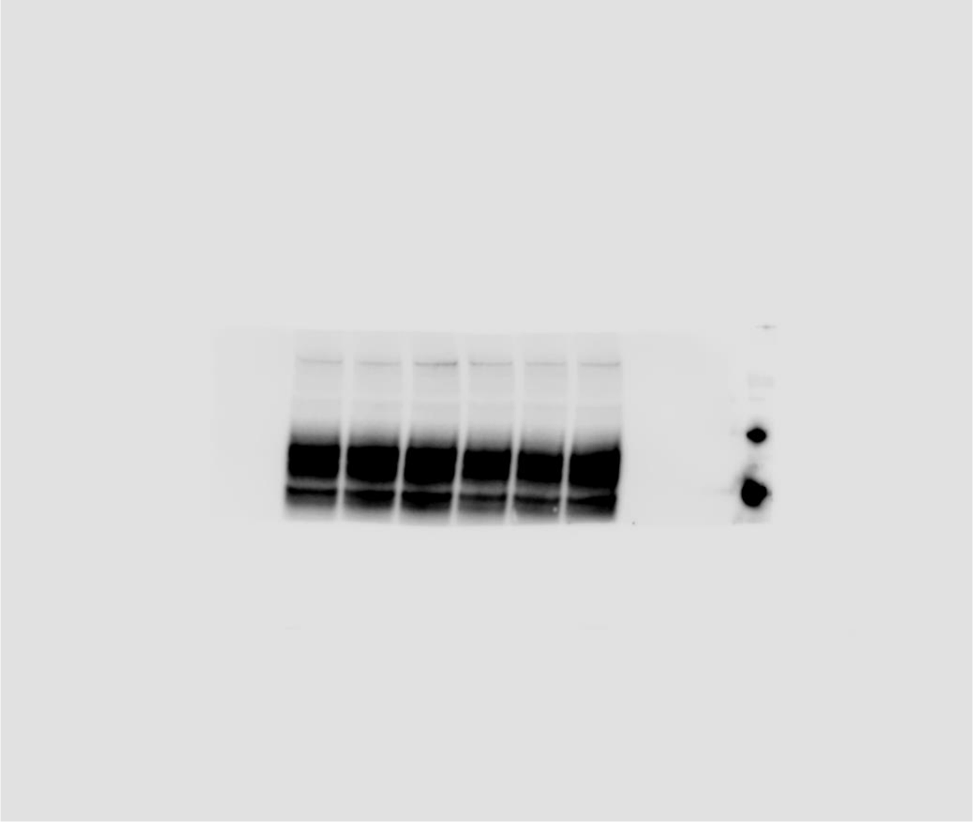

Figure 1A, second panel, uncropped image for WCLs, TRAM^{FLAG} (testing several lysates with TRAM^{FLAG} overexpression, lysate loaded to the first lane used for the IPs represented on the Fig. 1A). TRAM^{FLAG} in pCMV-(DYKDDDDK)-N vector used for transfection of HEK cells.

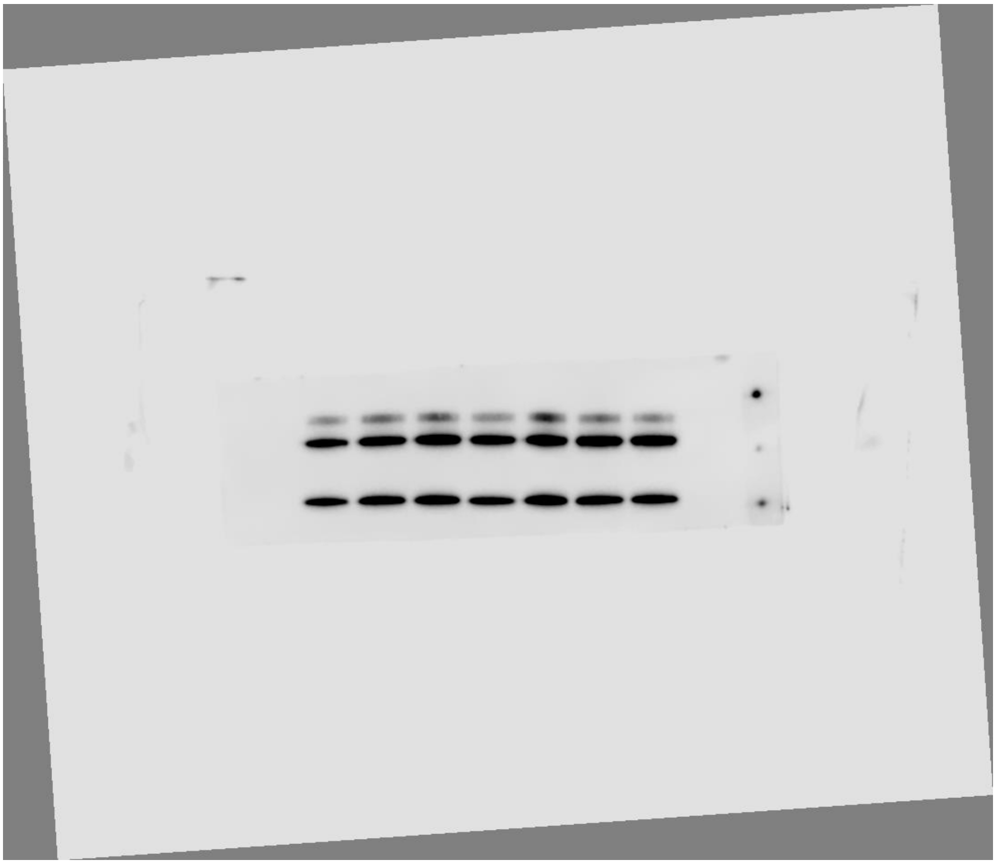

Figure 1A, third panel, uncropped image for anti-Flag IPs

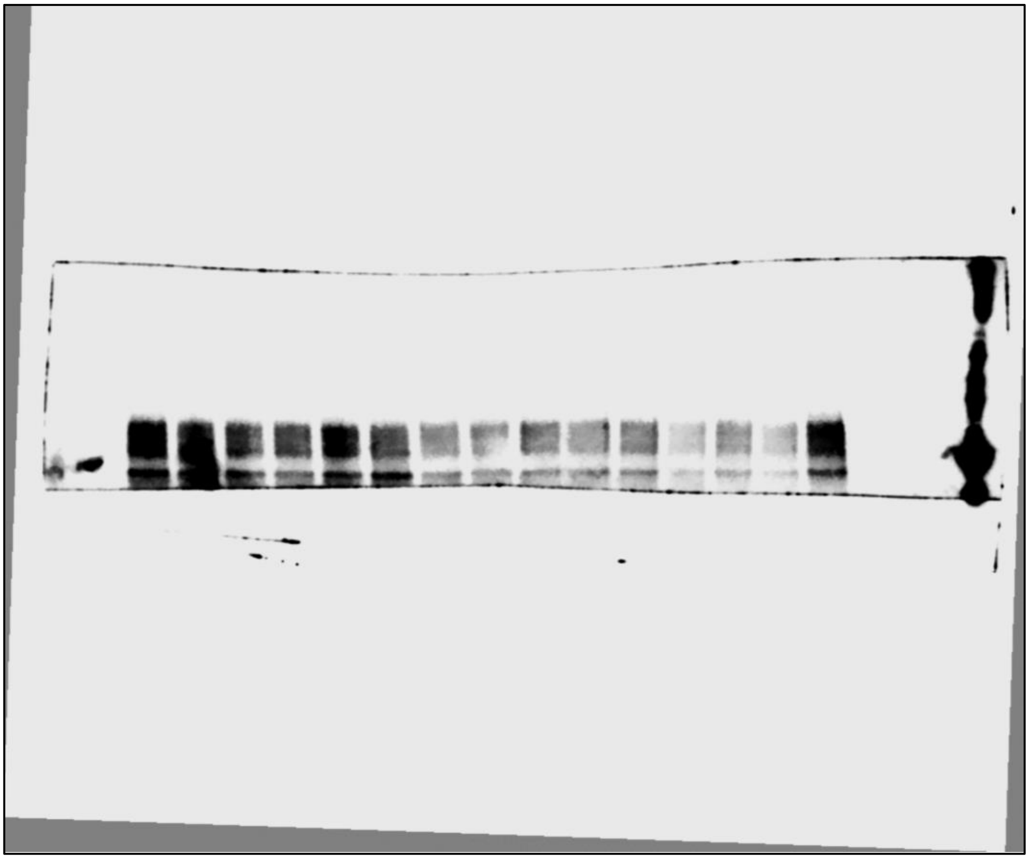

Figure 1A, fourth panel, uncropped image for anti-Flag IPs

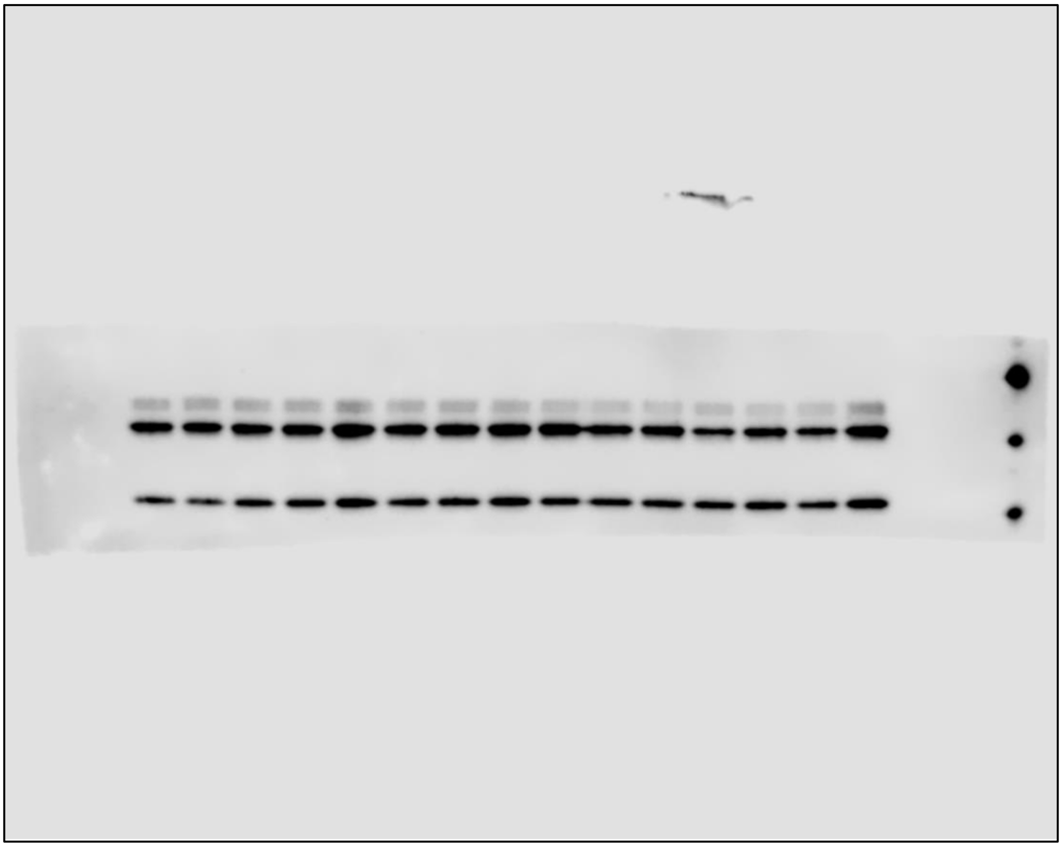

Figure 1B, TRAM YFP in the input (WCLs) and anti-Flag IPs, , cropped on the presented image

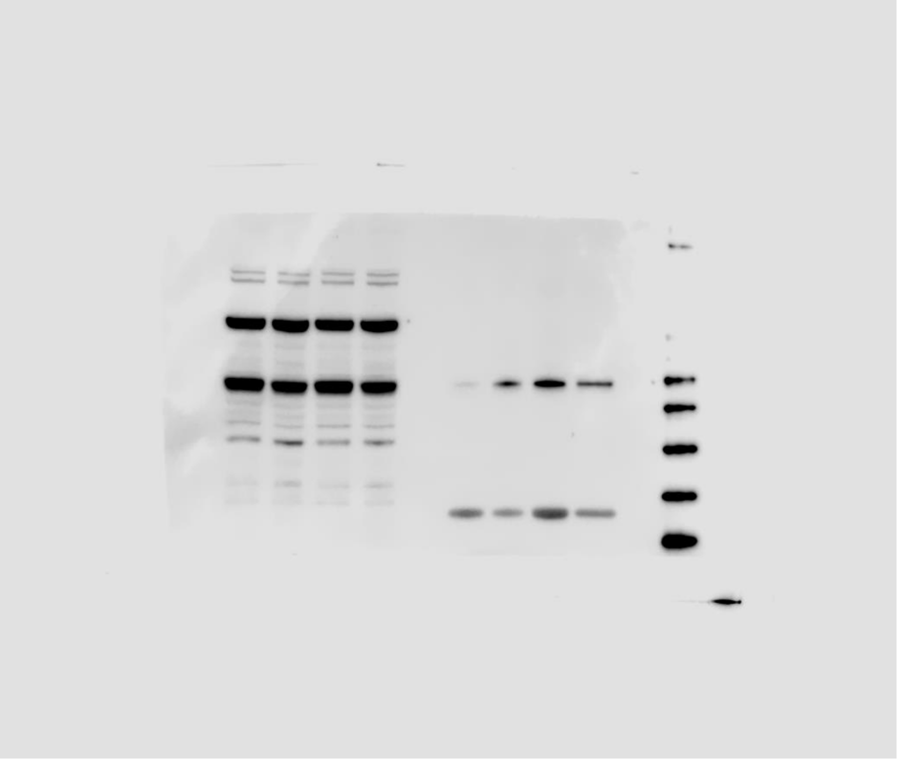

Figure 1B, left half represents SLAMF1^{FLAG} in the input/WCLs, cropped for the image

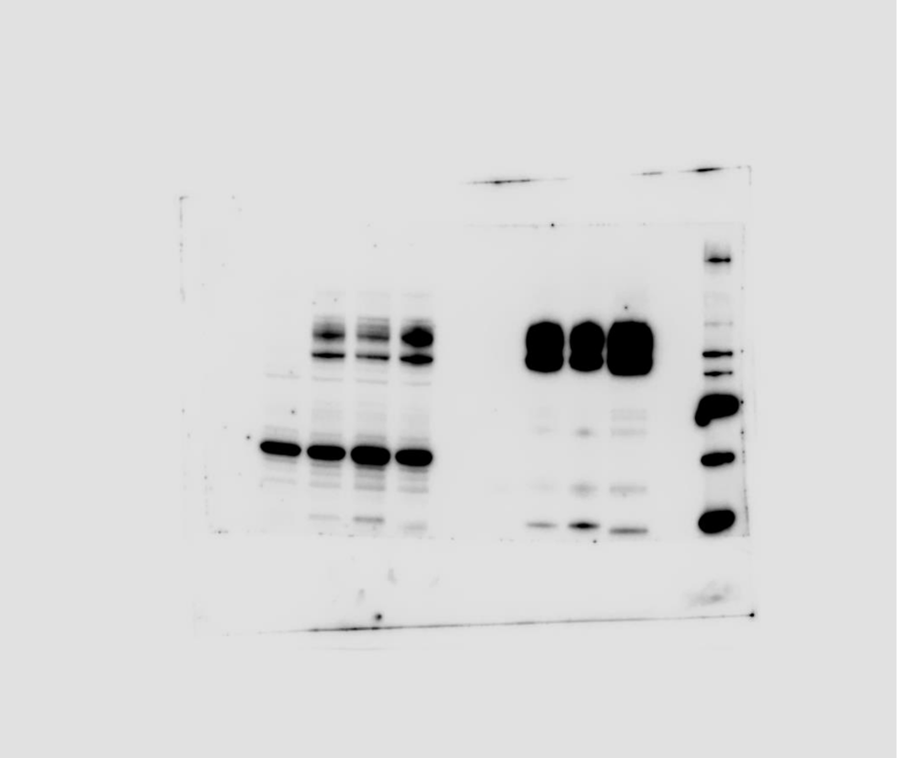

Figure 1B, SLAMF1^{FLAG} in the IPs (bottom right panel), cropped for the image

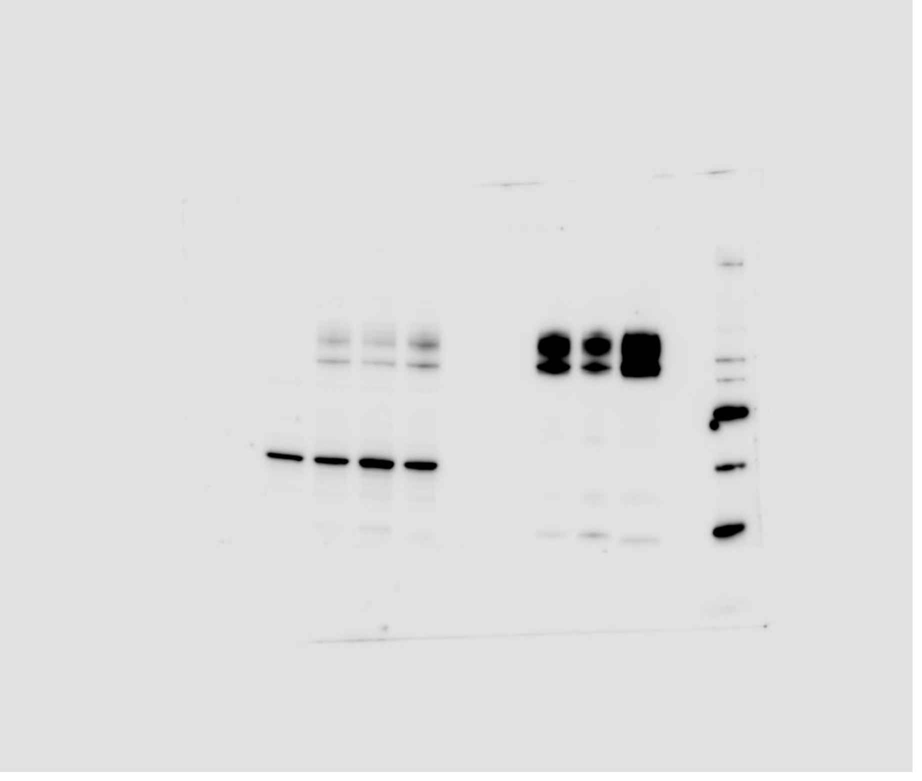

Figure 1D, upper panel, pSTAT1 on top, beta-tubulin on the same membrane

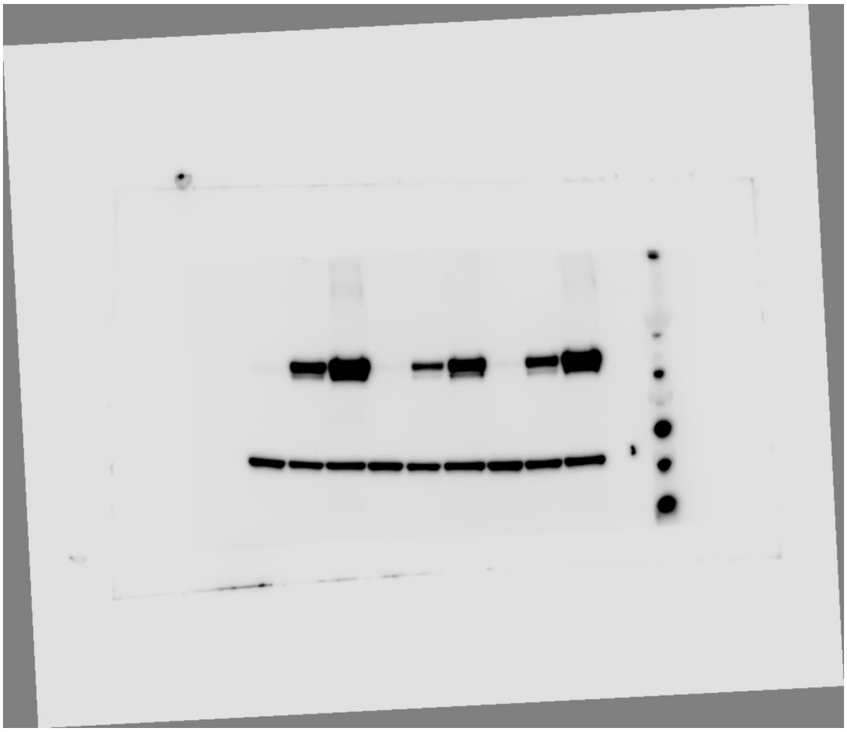

Figure 1D, middle panel, pSTAT1 on top, beta-tubulin on the same membrane

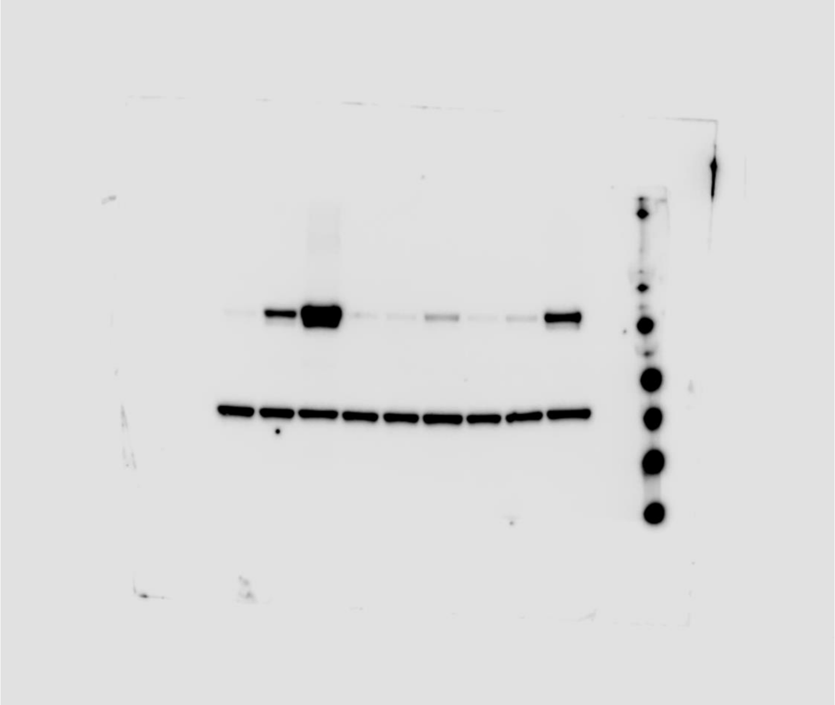

Figure 1D, lower (third) panel, pSTAT1 on top, beta-tubulin on the same membrane

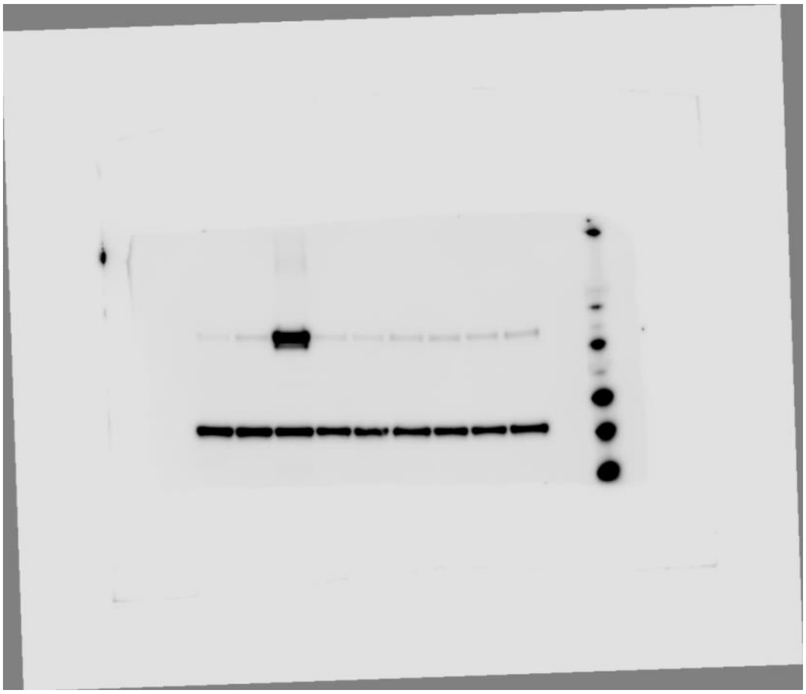

Supplement: Supplementary file 1 [file LSA-2023-02164_SdataF1.pdf]
