# Supplementary material for: Peptide derived from SLAMF1 prevents TLR4-mediated inflammation in vitro and in vivo
Source: Life Sci Alliance. 2023 Oct 3;6(12):e202302164. doi: 10.26508/lsa.202302164 (PMC10547912; doi:10.26508/lsa.202302164)
Supplement: Supplementary file 2 [file LSA-2023-02164_TableS1.docx]

**Table S1 - Sequences of peptides used in the screens for cytokines secretion and LDH release in THP-1 cells.**

| ***Peptide*** | ***Amino acid sequence*** |
| --- | --- |
| P7-Pen | ITVYASVTLT G RQIKIWFQNRRMKWKK |
| C3-Pen | IATYASTALT G RQIKIWFQNRRMKWKK |
| P10-Pen | ITVYASVTLP**E** G RQIKIWFQNRRMKWKK |
| P11-Pen | ITVYASVTL**TE** G RQIKIWFQNRRMKWKK |
| P6-Pen | ITVYASVTL**P** G RQIKIWFQNRRMKWKK |
| P7-A10-Pen | ITVYASVTL**A** G RQIKIWFQNRRMKWKK |
| P7-A1-Pen | **A**TVYASVTLT G RQIKIWFQNRRMKWKK |
| P7-A3-Pen | IT**A**YASVTLT G RQIKIWFQNRRMKWKK |
| P7-A4-Pen | ITV**A**ASVTLT G RQIKIWFQNRRMKWKK |
| P7-A6-Pen | ITVYA**A**VTLT G RQIKIWFQNRRMKWKK |
| P7-A7-Pen | ITVYAS**A**TLT G RQIKIWFQNRRMKWKK |
| P7-A8-Pen | ITVYASV**A**LT G RQIKIWFQNRRMKWKK |
| P7-A9-Pen | ITVYASVT**A**T G RQIKIWFQNRRMKWKK |
| P7-G9-Pen | ITVYASVT**G**T G RQIKIWFQNRRMKWKK |
| P7-I9-Pen | ITVYASVT**I**T G RQIKIWFQNRRMKWKK |
| P7-L3-Pen | IT**L**YASVTLT G RQIKIWFQNRRMKWKK |
| P7-L5-Pen | ITVY**L**SVTLT G RQIKIWFQNRRMKWKK |
| P7-N4-Pen | ITV**N**ASVTLT G RQIKIWFQNRRMKWKK |
| P7-S10-Pen | ITVYASVTL**S** G RQIKIWFQNRRMKWKK |
| P7-S2-Pen | I**S**VYASVTLT G RQIKIWFQNRRMKWKK |
| P7-T3-Pen | IT**T**YASVTLT G RQIKIWFQNRRMKWKK |
| P7-T4-Pen | ITV**T**ASVTLT G RQIKIWFQNRRMKWKK |
| P7-V10-Pen | ITVYASVTL**V** G RQIKIWFQNRRMKWKK |
| P7-V4-Pen | ITV**V**ASVTLT G RQIKIWFQNRRMKWKK |
| Penetratin | RQIKIWFQNRRMKWKK |
