# Supplementary material for: Peptide derived from SLAMF1 prevents TLR4-mediated inflammation in vitro and in vivo
Source: Life Sci Alliance. 2023 Oct 3;6(12):e202302164. doi: 10.26508/lsa.202302164 (PMC10547912; doi:10.26508/lsa.202302164)

Source file for Figure 2

Peptide derived from SLAMF1 prevents TLR4-mediated inflammation *in vitro* and *in vivo*

Figure 2C, top panel, uncropped image for pSTAT1 – parts of this blot shown on the figure 2C

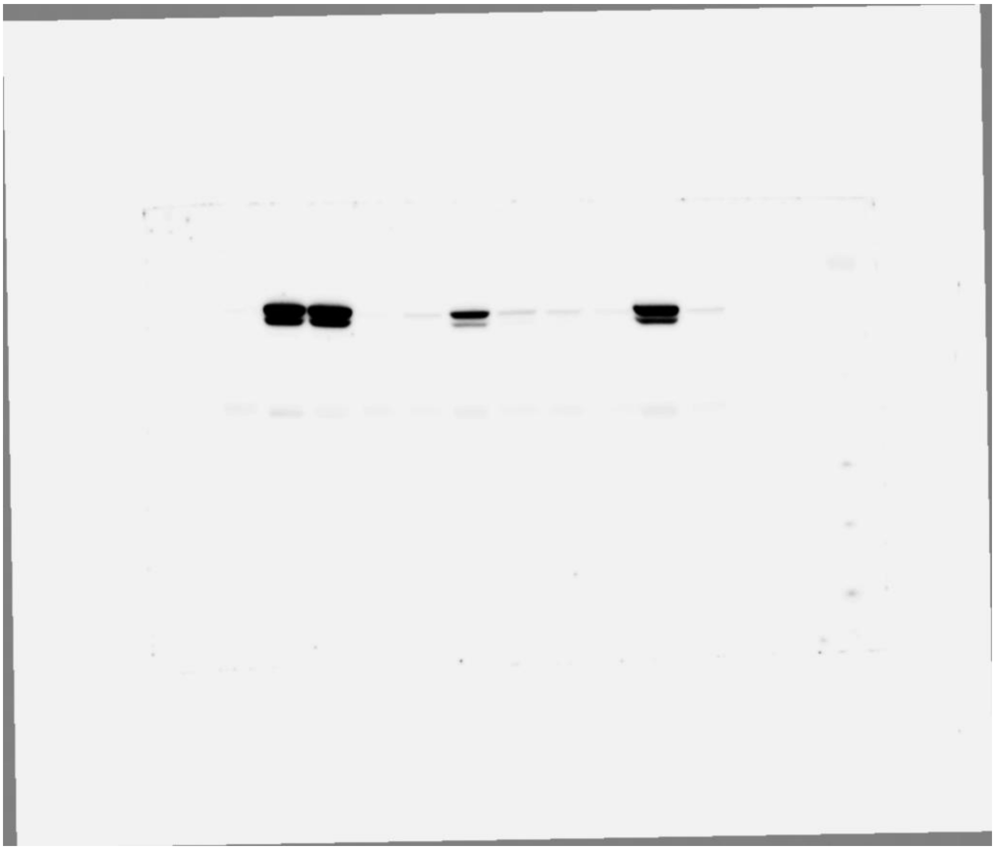

Figure 2C, lowerpanel, uncropped image for beta-tubulin – parts of this blot shown on the figure 2C

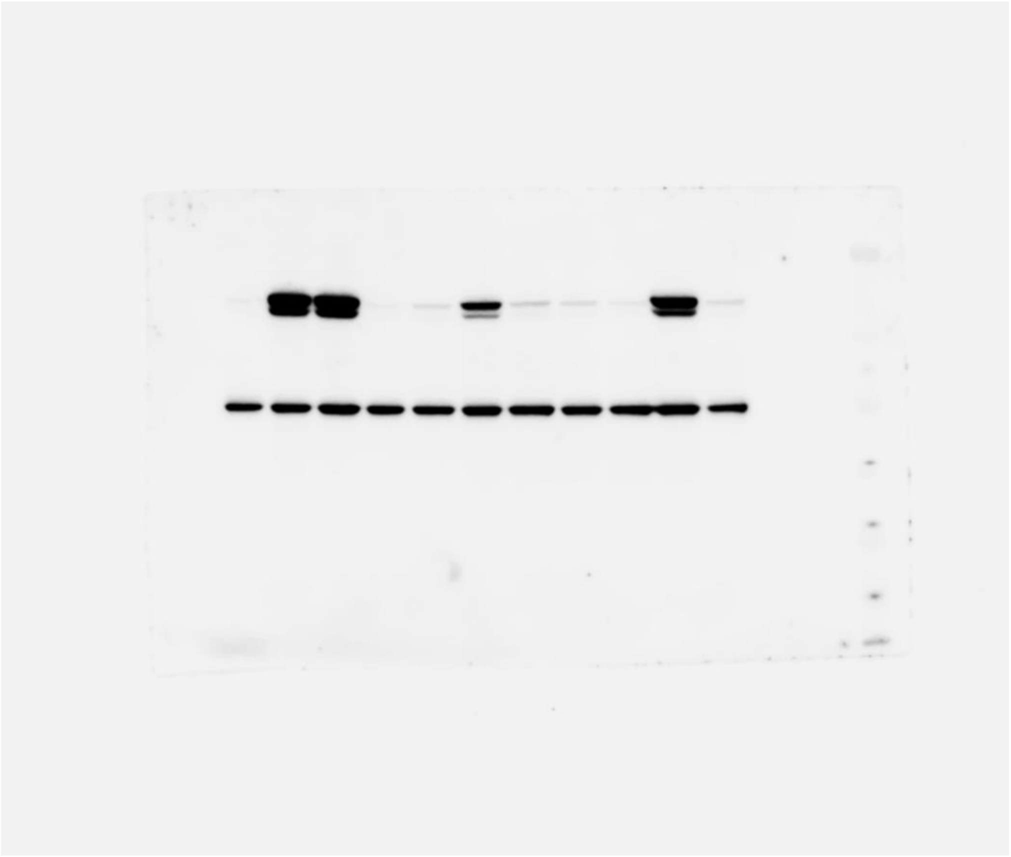

Supplement: Supplementary file 3 [file LSA-2023-02164_SdataF2.pdf]
