# Supplementary material for: Peptide derived from SLAMF1 prevents TLR4-mediated inflammation in vitro and in vivo
Source: Life Sci Alliance. 2023 Oct 3;6(12):e202302164. doi: 10.26508/lsa.202302164 (PMC10547912; doi:10.26508/lsa.202302164)

Source file for Figure 4

Peptide derived from SLAMF1 prevents TLR4-mediated inflammation *in vitro* and *in vivo*

Figure 4A, SLAMF1 WB for WCLs/input

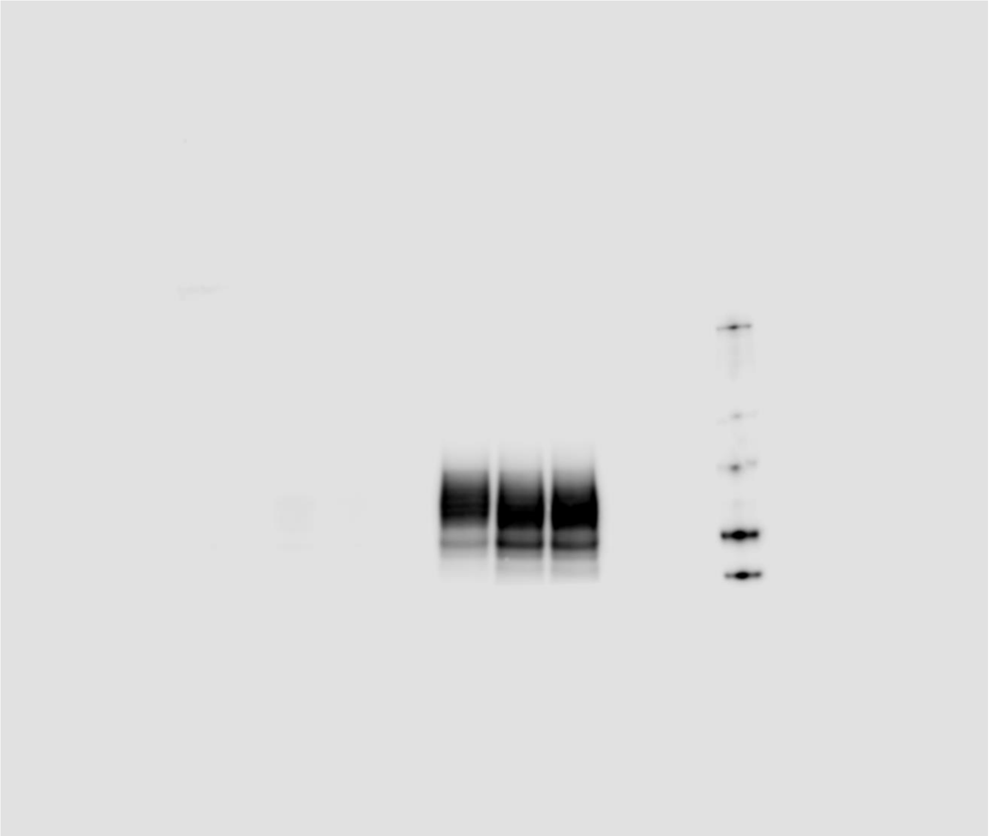

Figure 4A, SLAMF1 WB for IPs

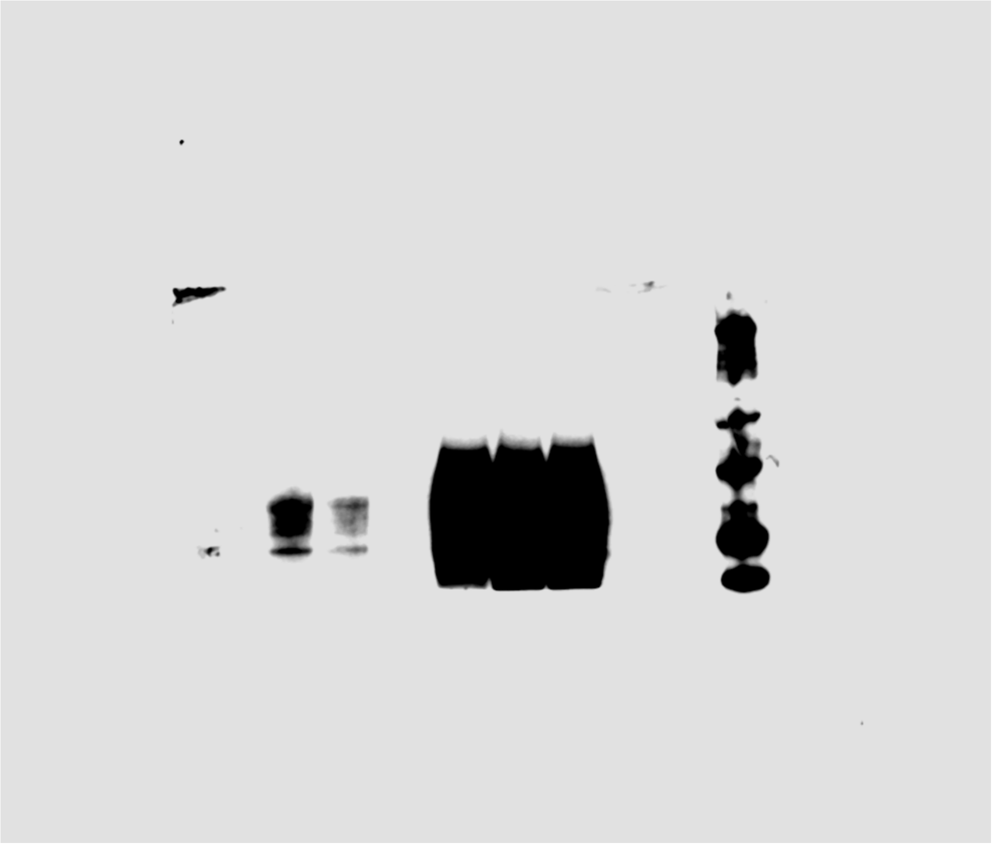

Figure 4A, TRAM FLAG WB for WCLs/input on the left and IPs on the right, presented as cropped images to exclude empty lanes on the figure 4C

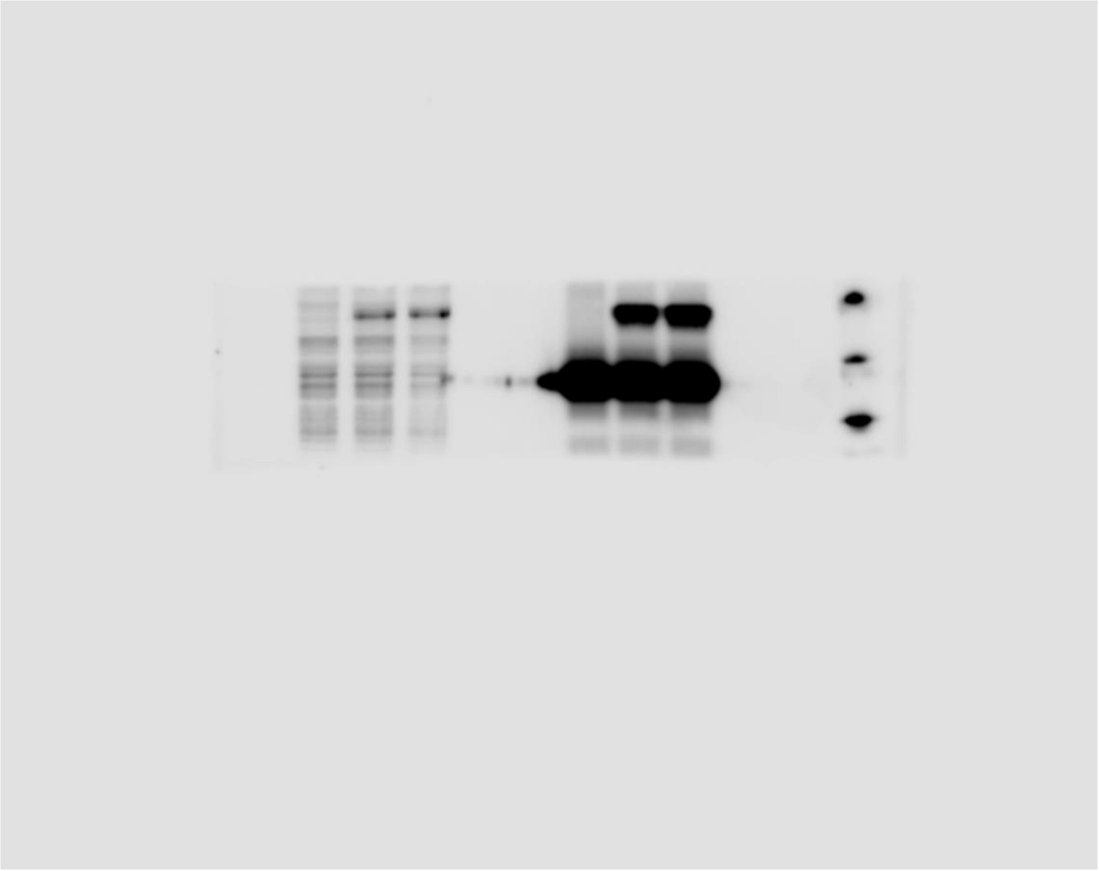

Figure 4B, TRAM^{YFP} WB for WCLs/input and IPs

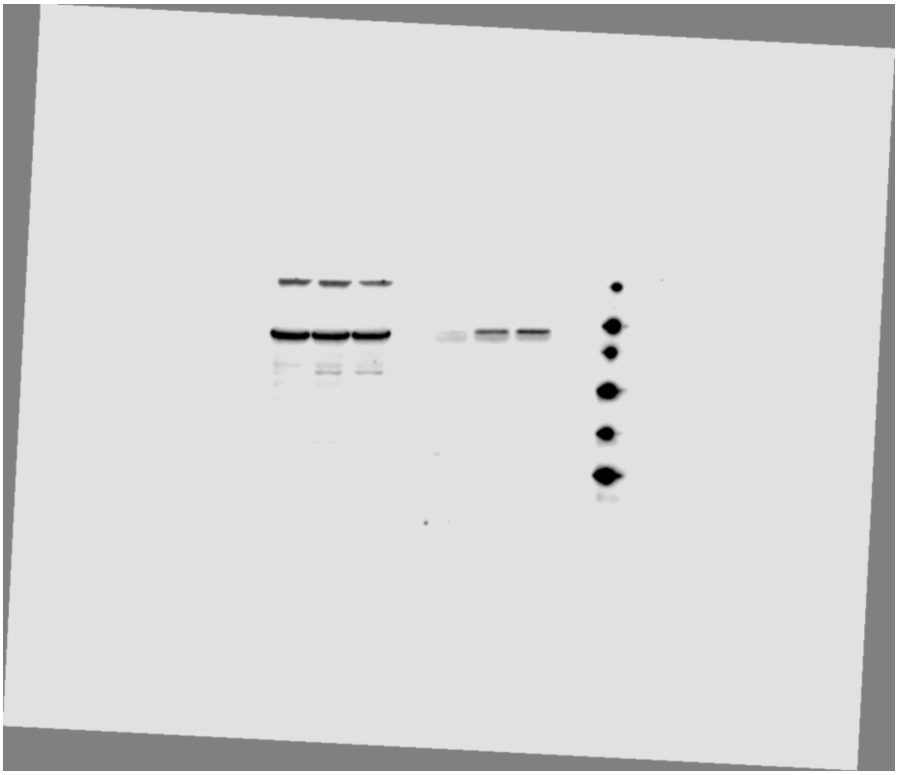

Figure 4B, TLR4^{FLAG} WB for WCLs/input and IPs

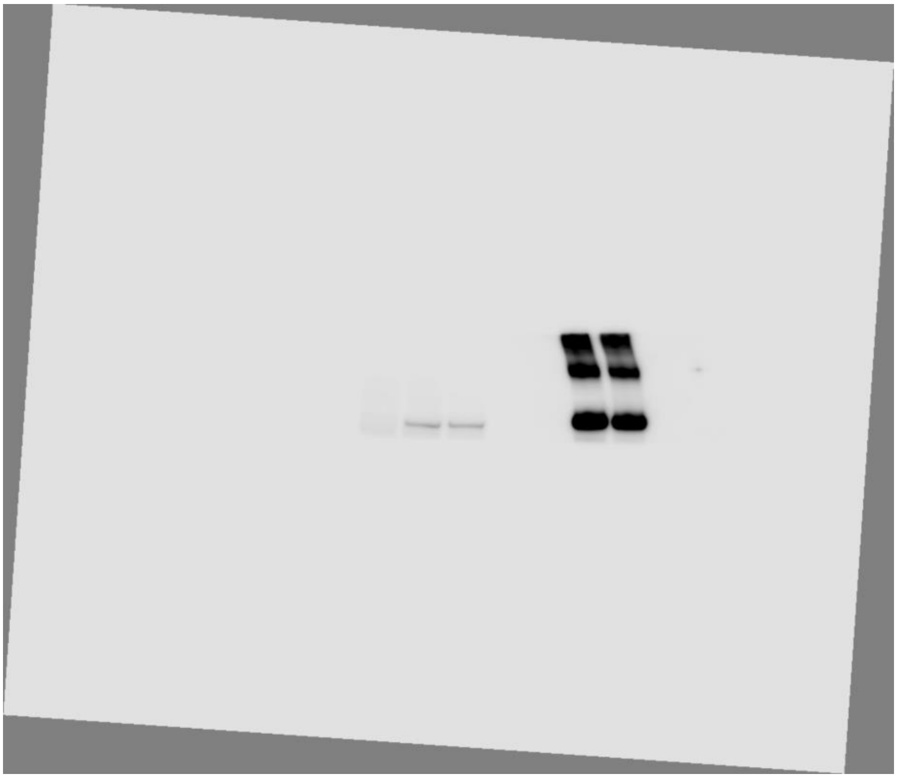

Figure 4C, TRIF^{HA} WB for WCLs/input and IPs

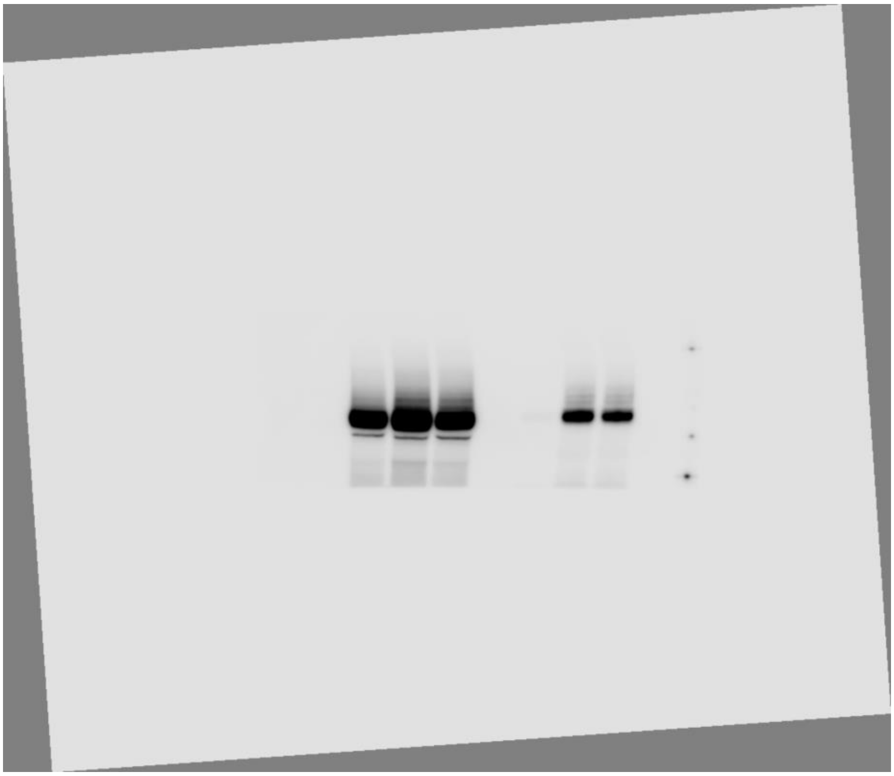

Figure 4C, TRAM^{FLAG} WB for WCLs/input and IPs

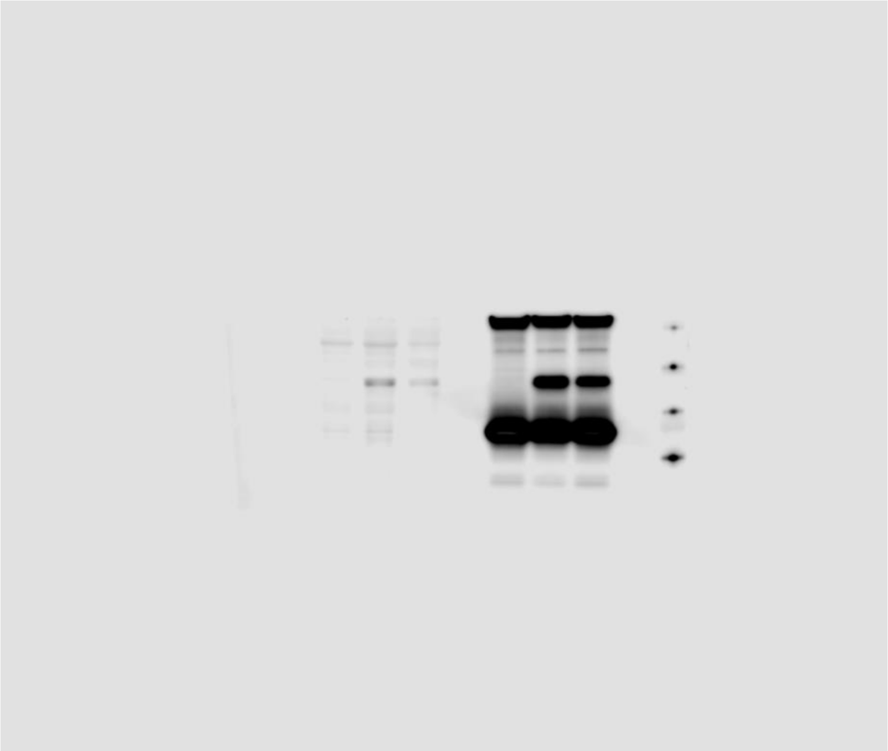

Figure 4D, FIP2^{EGFP} WB for WCLs/input

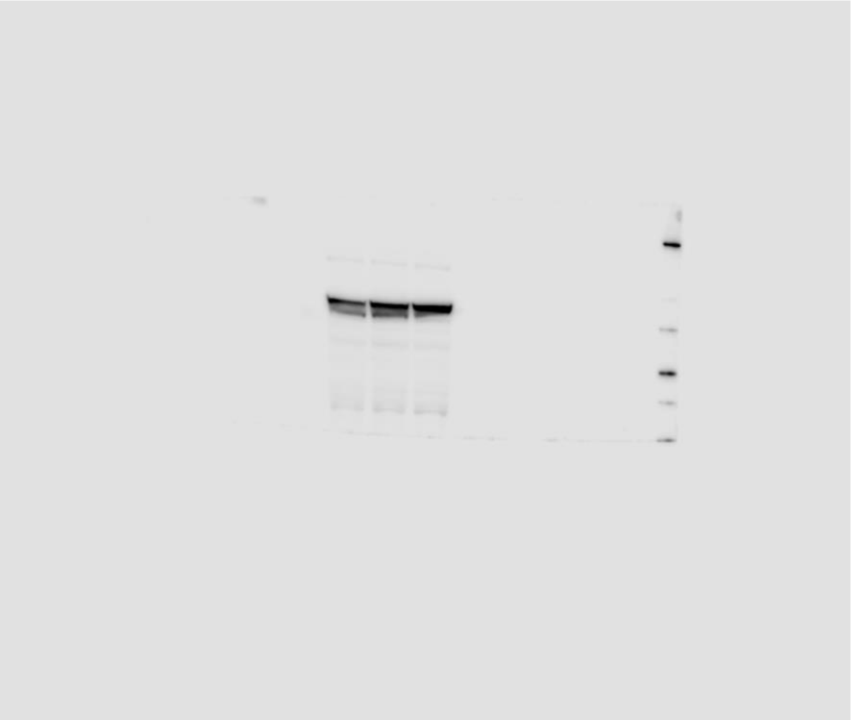

Figure 4D, FIP2^{EGFP} WB for IPs

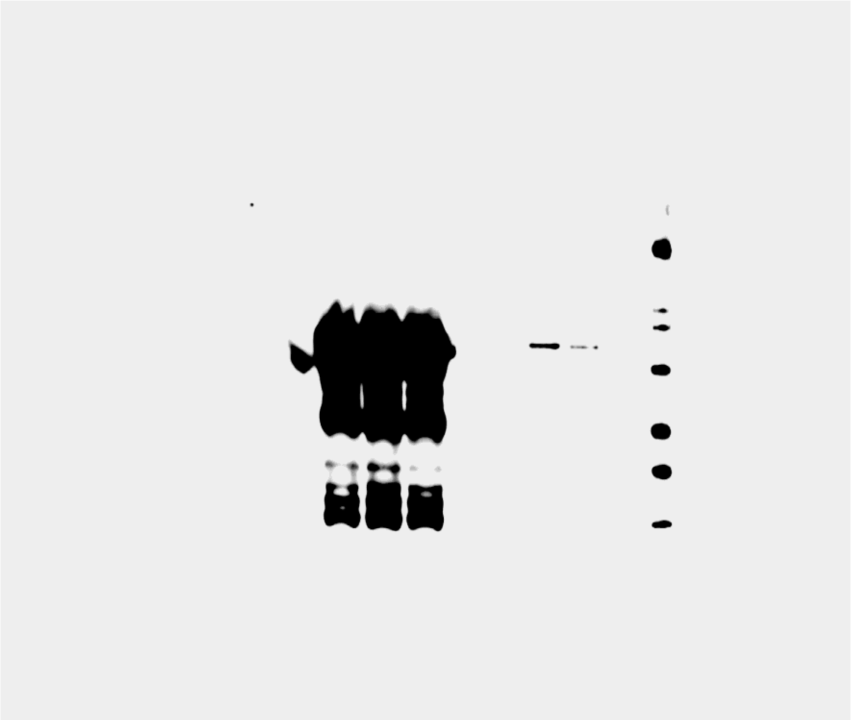

Figure 4D, TRAM^{FLAG} WB for WCLs/input and IPs, empty lane cropped on the images presented in figure 4D lower panels

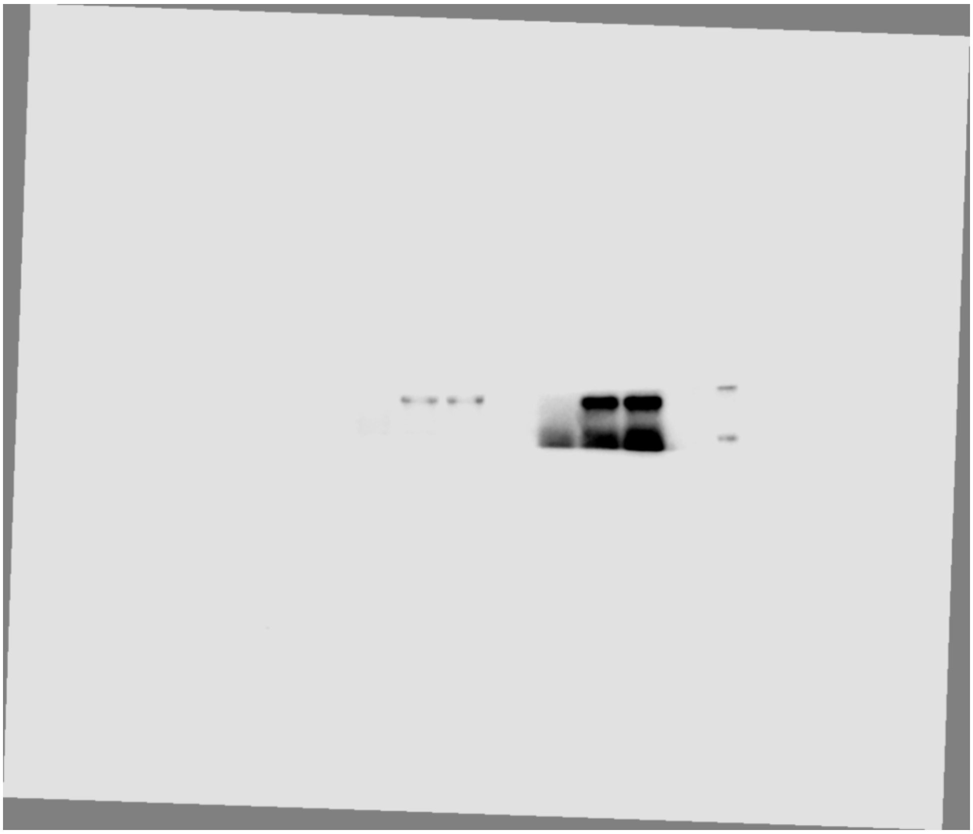

Supplement: Supplementary file 4 [file LSA-2023-02164_SdataF4.pdf]
