# Supplementary material for: Peptide derived from SLAMF1 prevents TLR4-mediated inflammation in vitro and in vivo
Source: Life Sci Alliance. 2023 Oct 3;6(12):e202302164. doi: 10.26508/lsa.202302164 (PMC10547912; doi:10.26508/lsa.202302164)

Source file for Figure 5

Peptide derived from SLAMF1 prevents TLR4-mediated inflammation *in vitro* and *in vivo*

Figure 5B fluorescent image

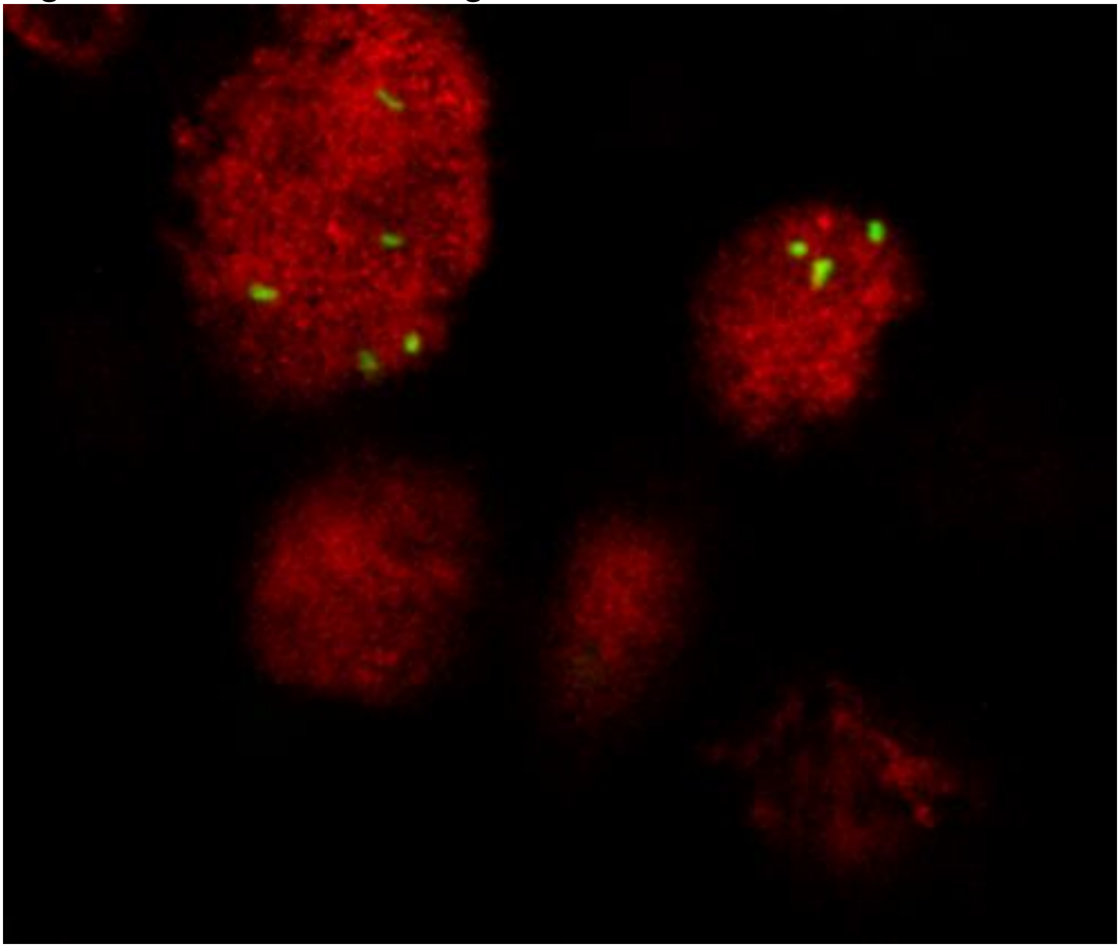

Figure 5B, bright field image

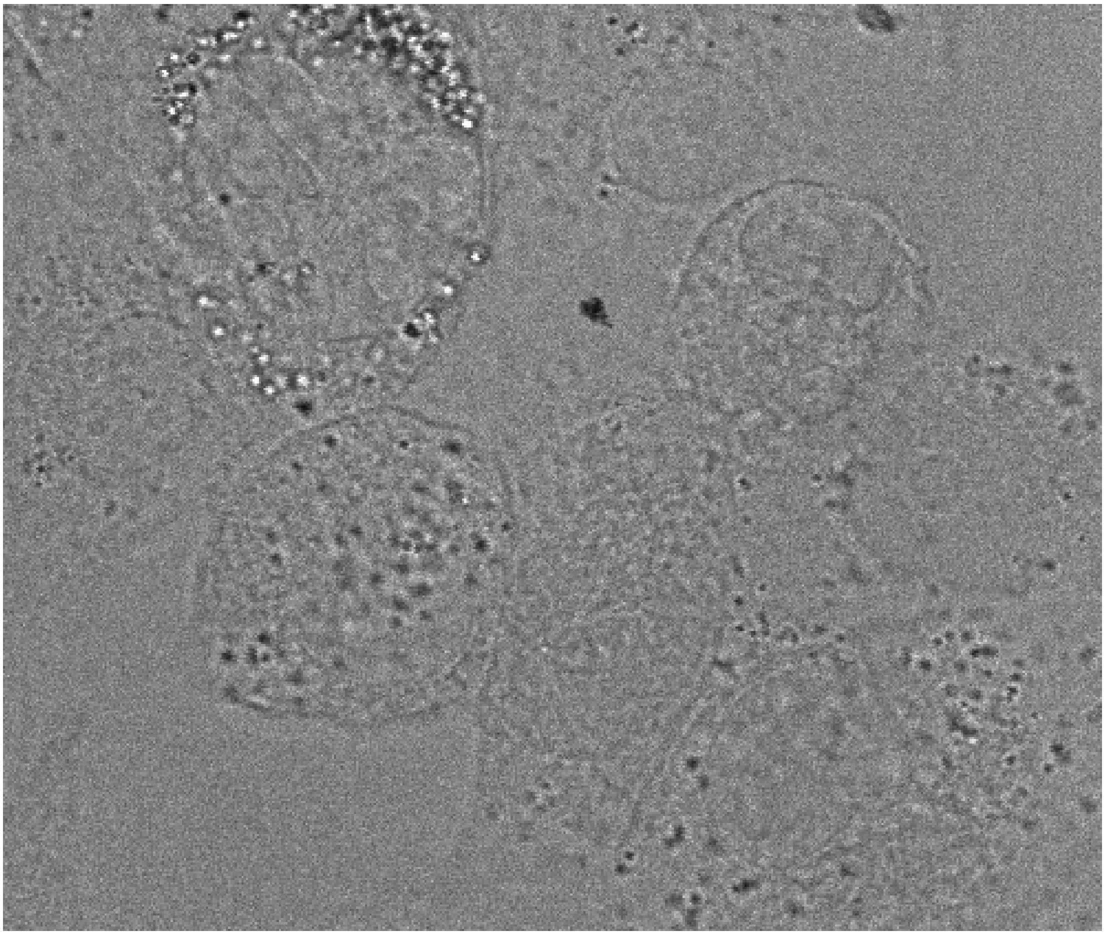

Figure 5E fluorescent image

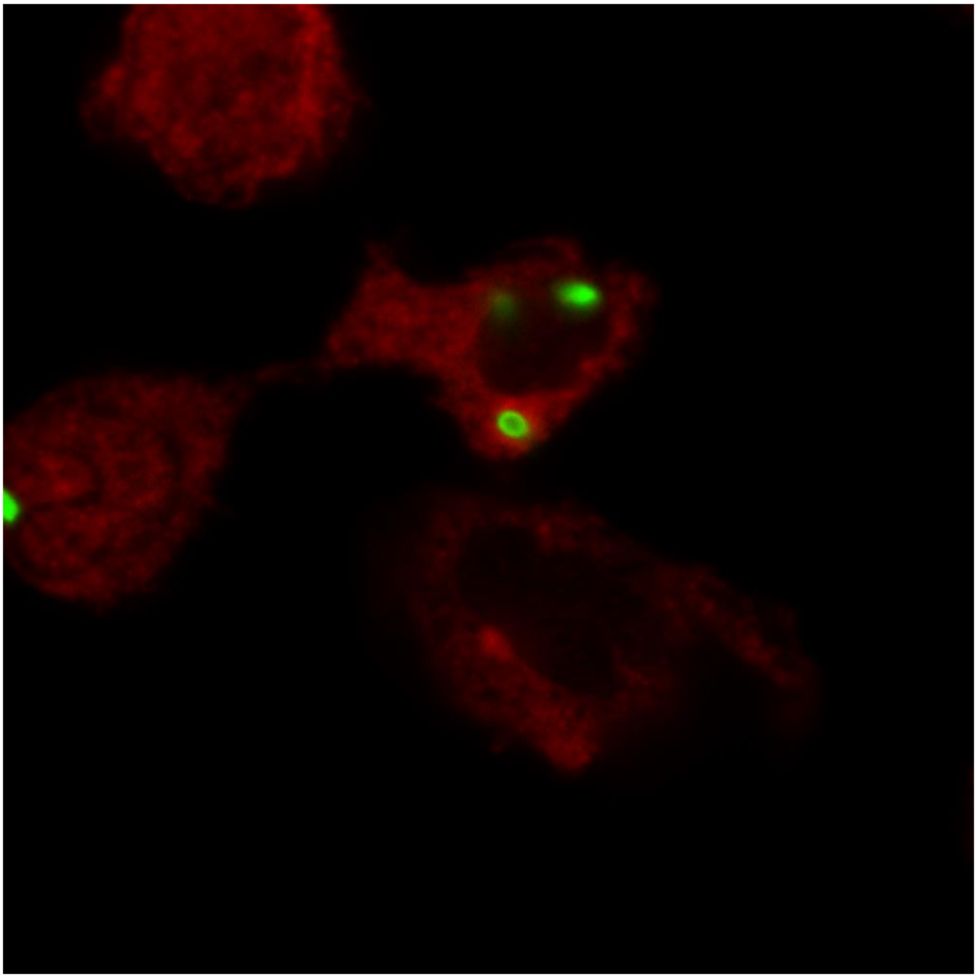

Figure 5E, bright field image

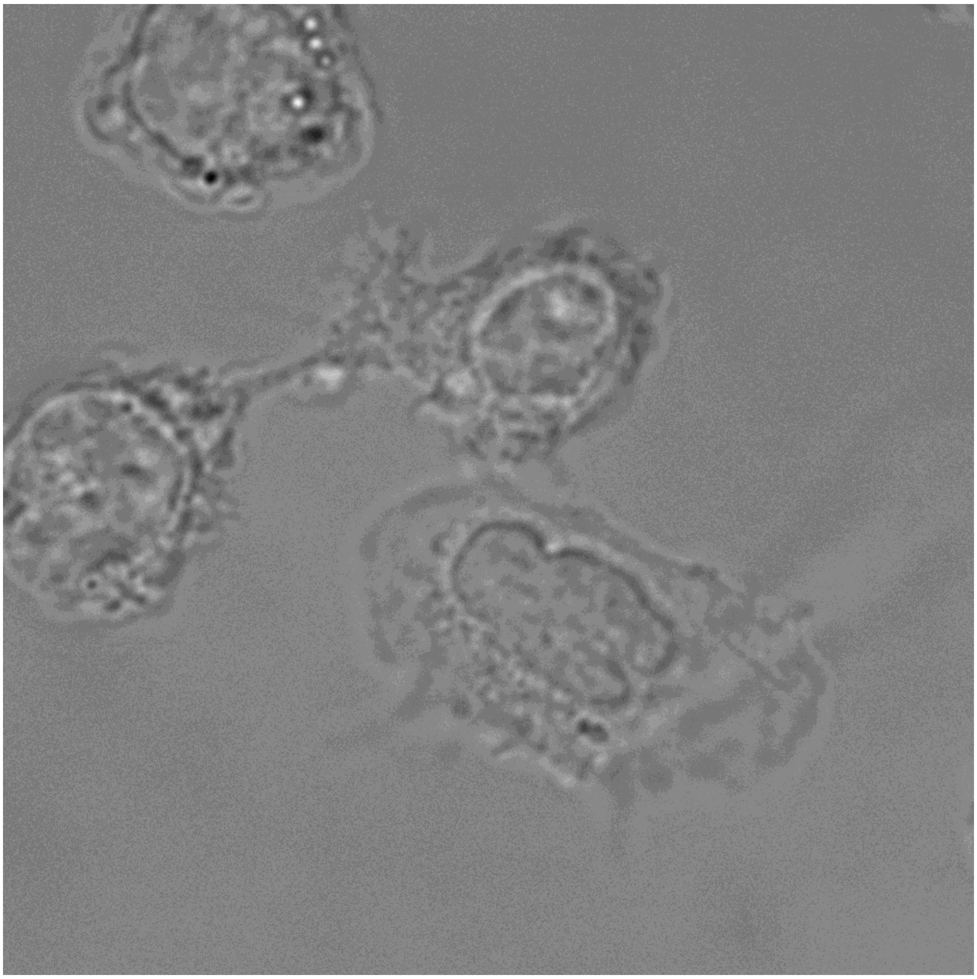

Supplement: Supplementary file 5 [file LSA-2023-02164_SdataF5.pdf]
