# Supplementary material for: Peptide derived from SLAMF1 prevents TLR4-mediated inflammation in vitro and in vivo
Source: Life Sci Alliance. 2023 Oct 3;6(12):e202302164. doi: 10.26508/lsa.202302164 (PMC10547912; doi:10.26508/lsa.202302164)

Source file for Figure 6

Peptide derived from SLAMF1 prevents TLR4-mediated inflammation *in vitro* and *in vivo*

Figure 6B, first panel for TRAM

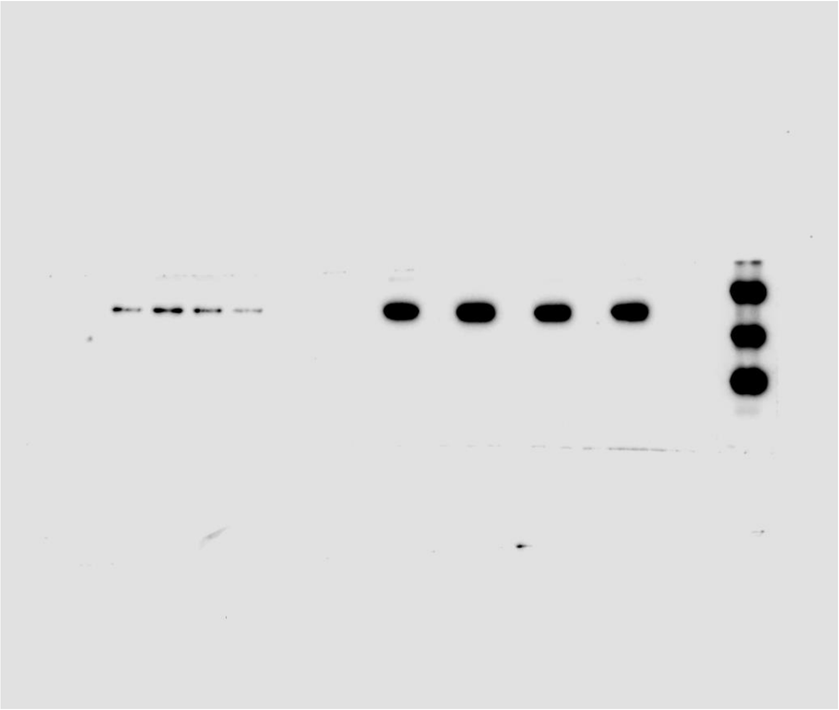

Figure 6B, second panel for TIRAP

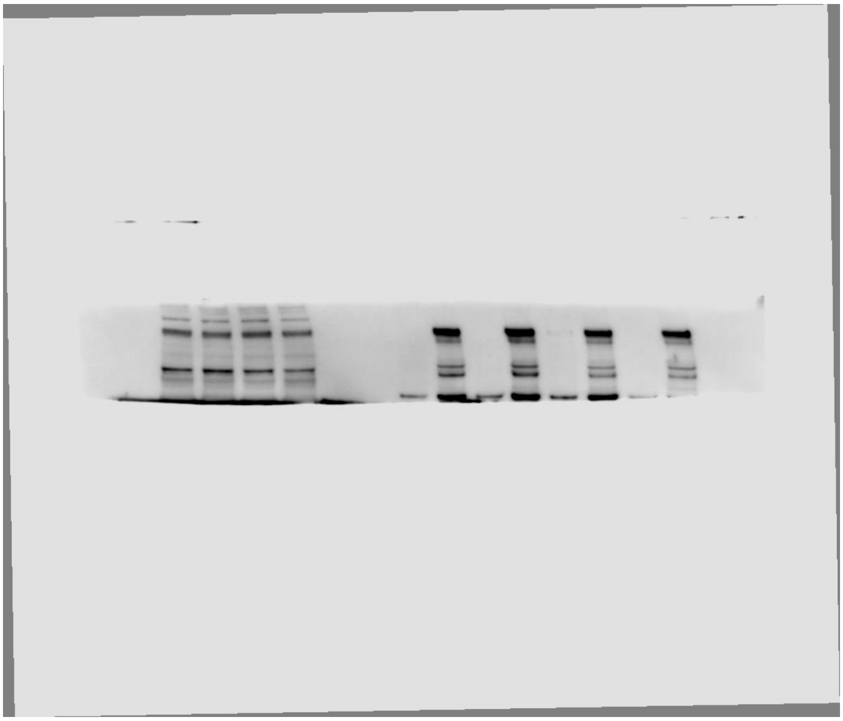

Figure 6B, third panel for IRAK1

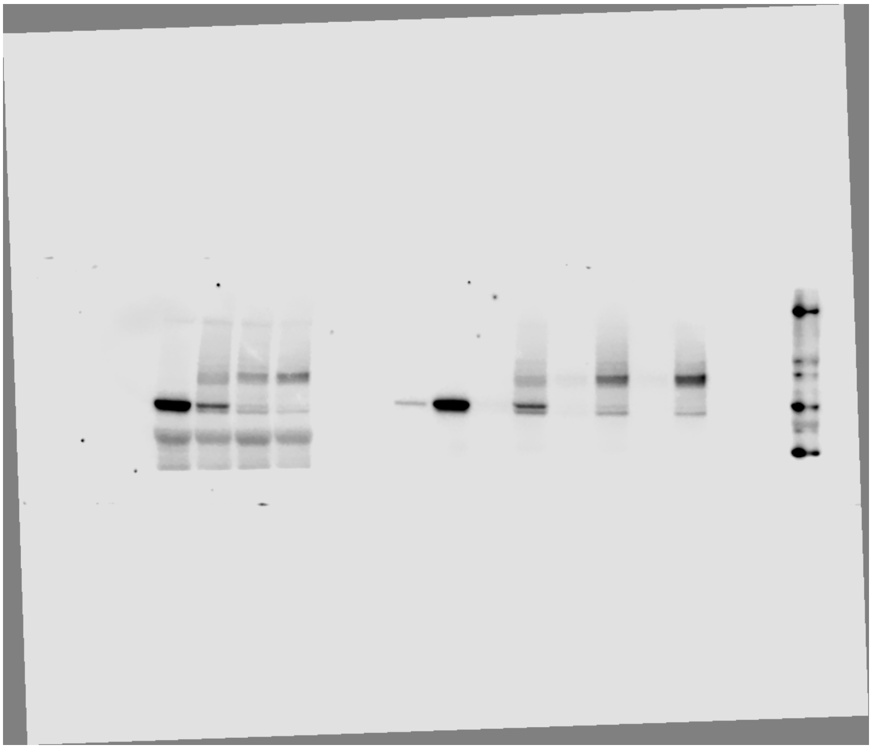

Figure 6B, fourth panel for IRAK4

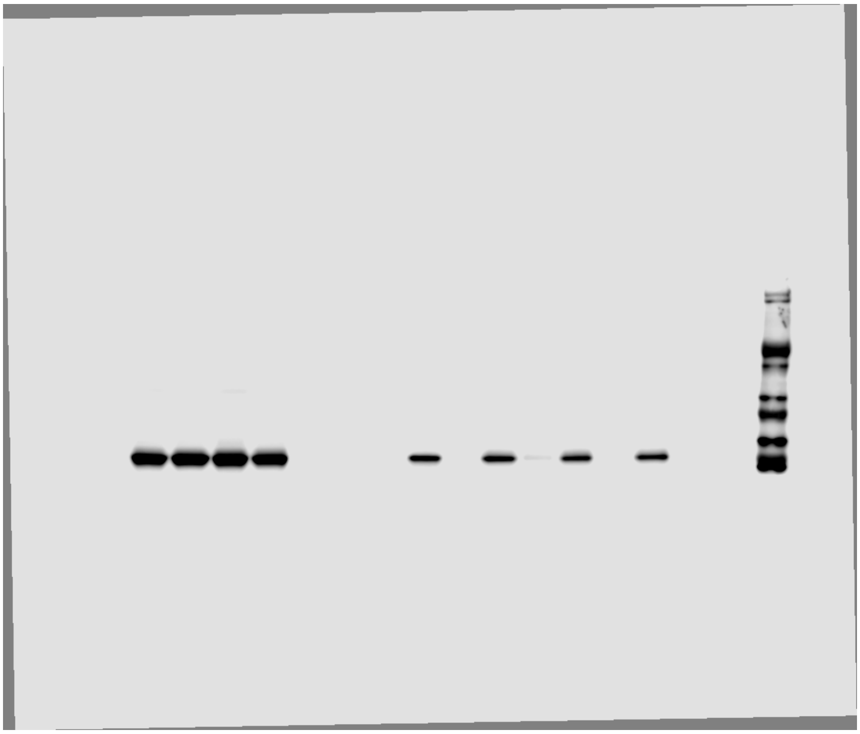

Figure 6B, fifth panel for MyD88

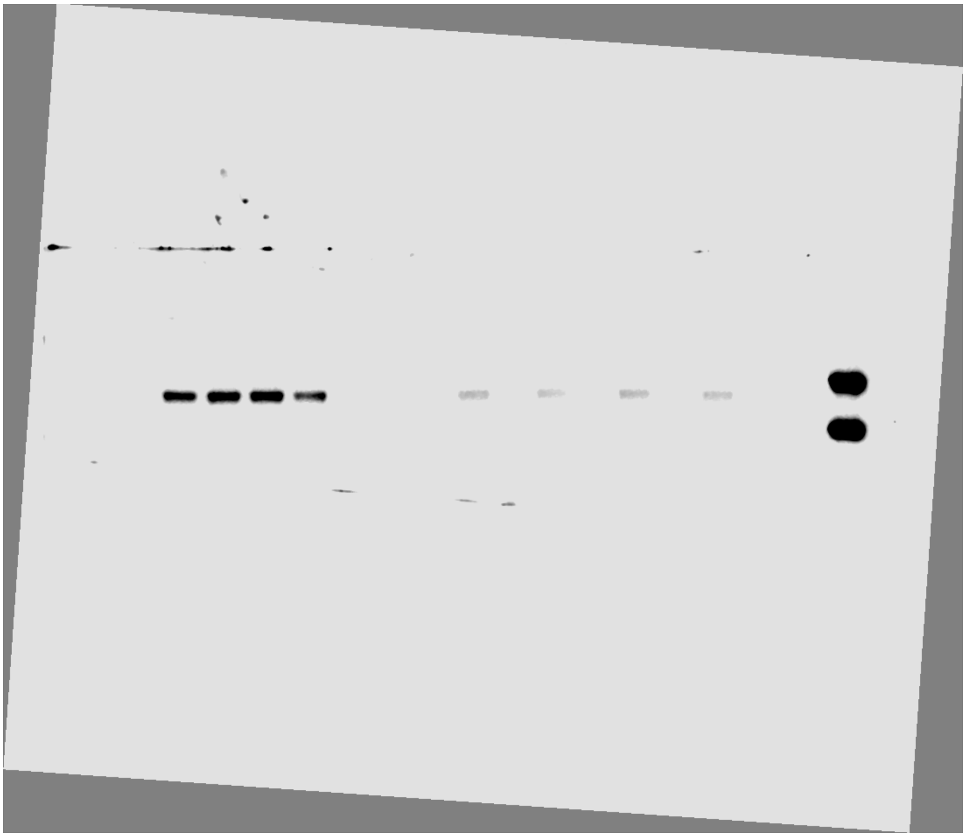

Figure 6B, six panel for stained gel (Image Lab, BioRad)

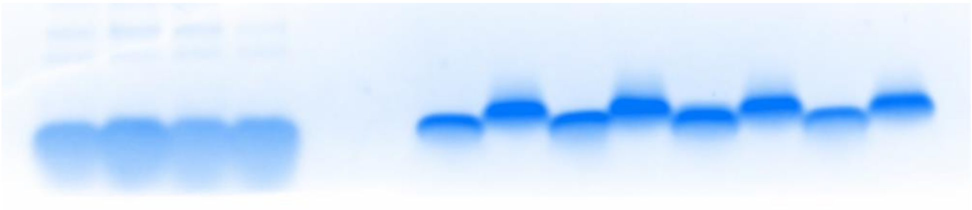

Figure 6C, upper panel for IRAK4

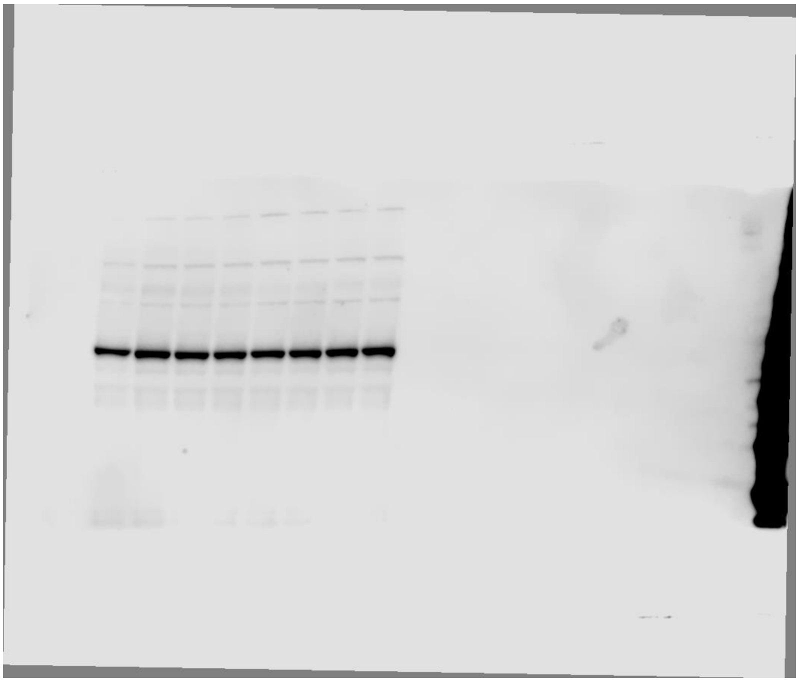

Figure 6C, middle panel for IRAK1

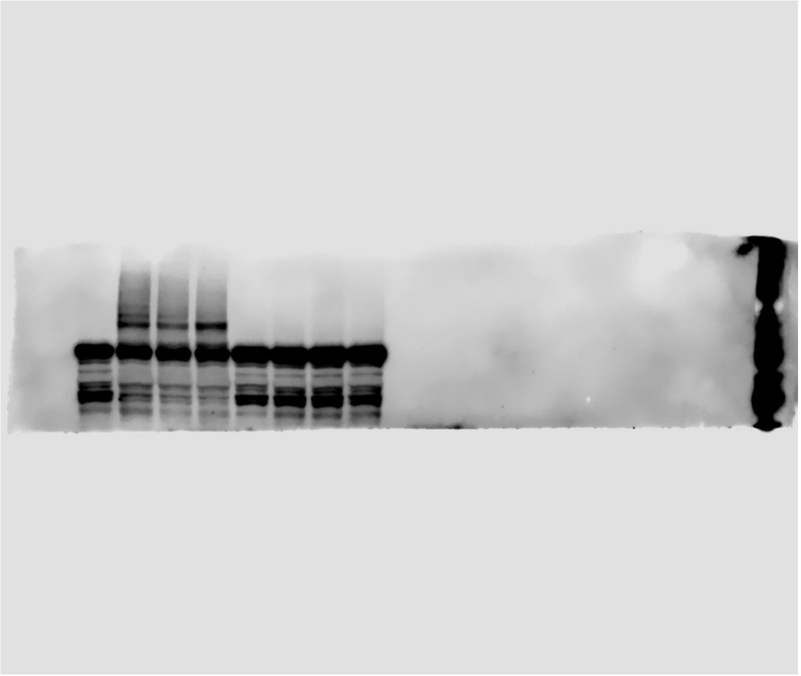

Figure 6C, middle panel for MyD88

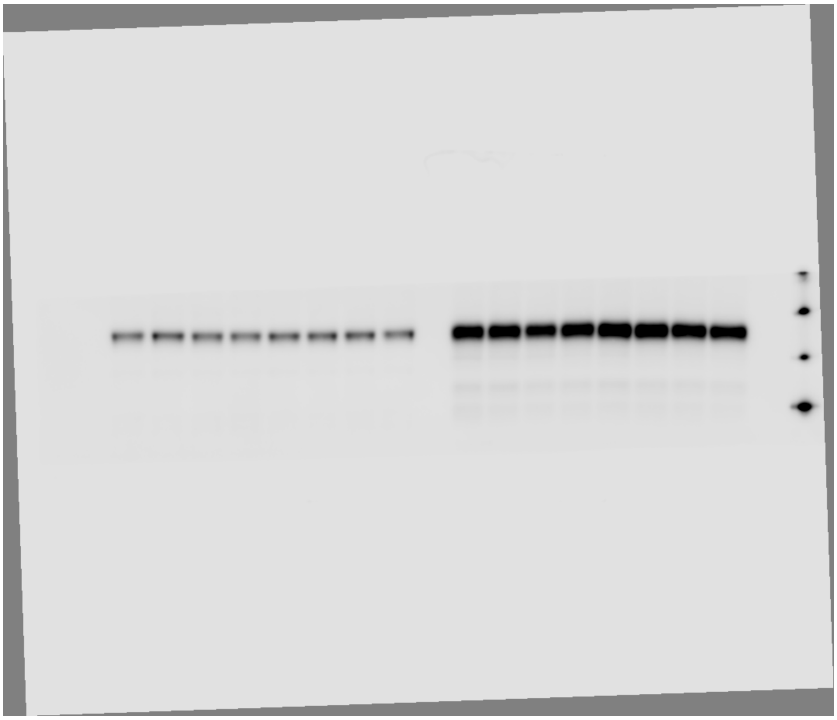

Figure 6D, top panel for p-p38 MAPK

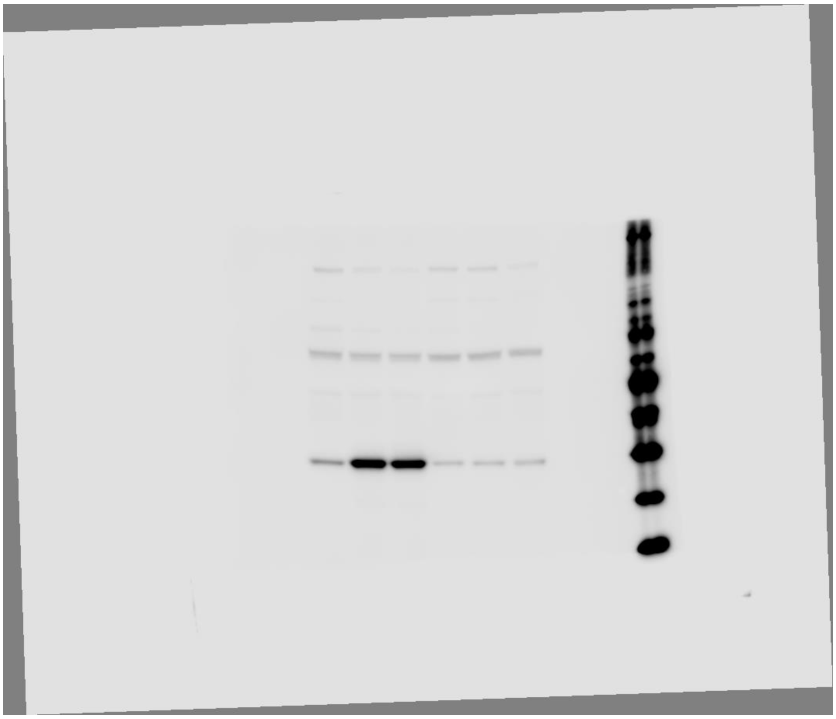

Figure 6D, bottom panel for beta-tubulin

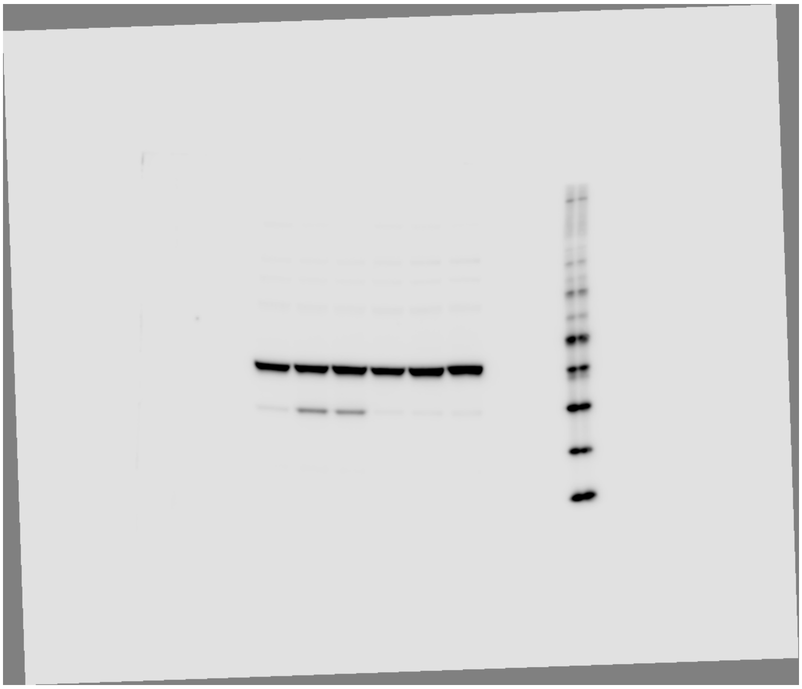

Figure 6E, MyD88 in WCLs/input and IPs

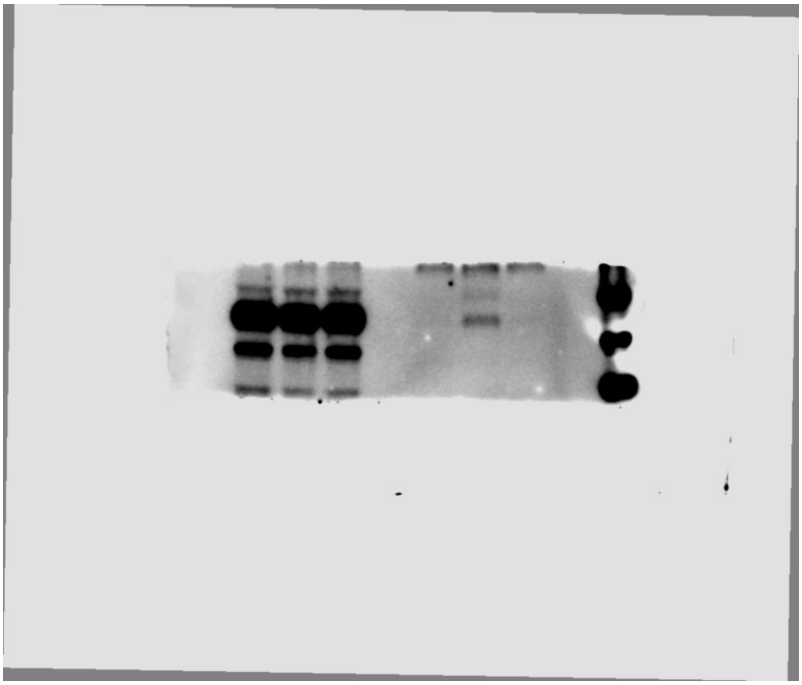

Figure 6E, bottom left panel for IRAK1 in lysates

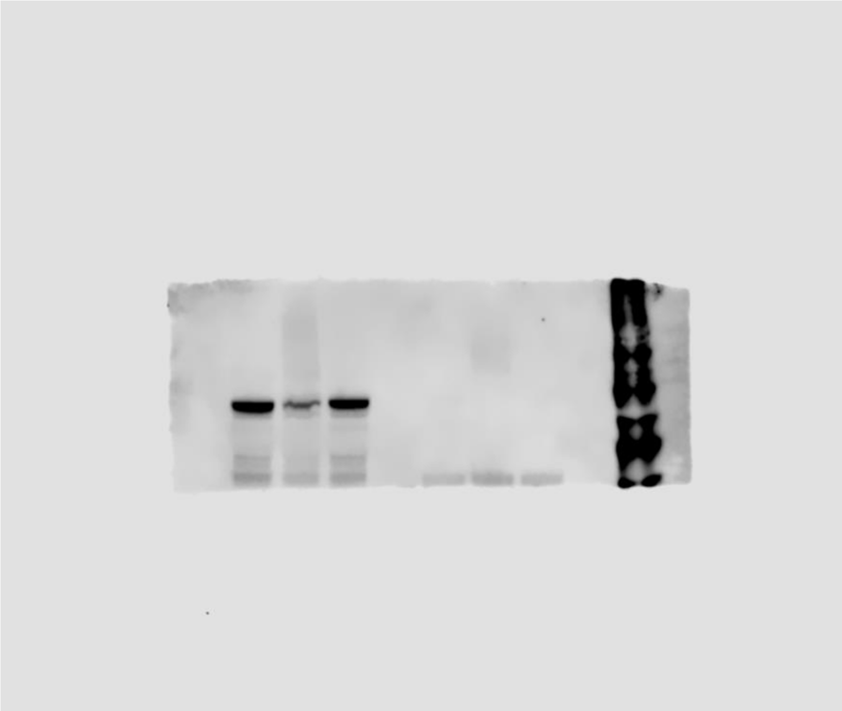

Figure 6E, bottom right panel for IRAK1 in IPs

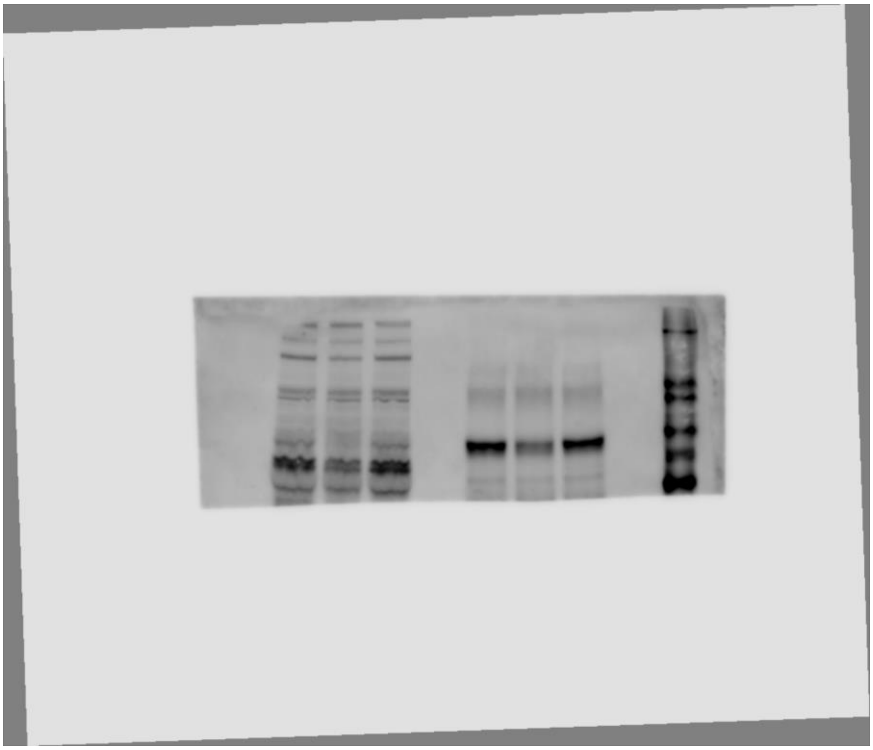

Figure 6F, top panel for MyD88 in WCLs and IPs

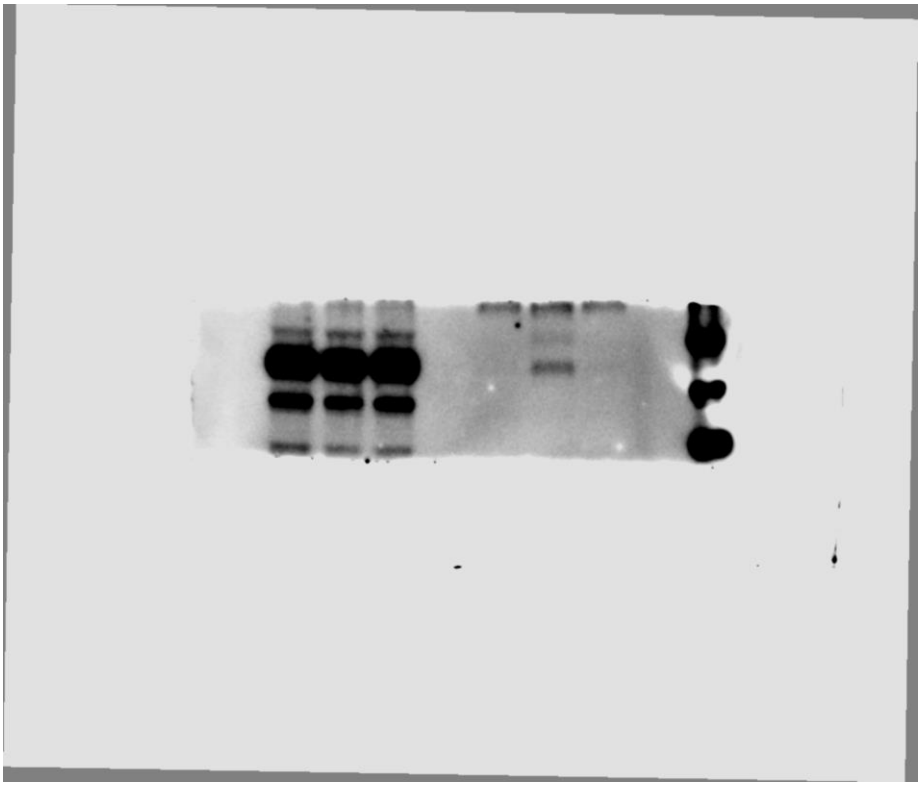

Figure 6F, left middle panel for IRAK1 in WCLs/input

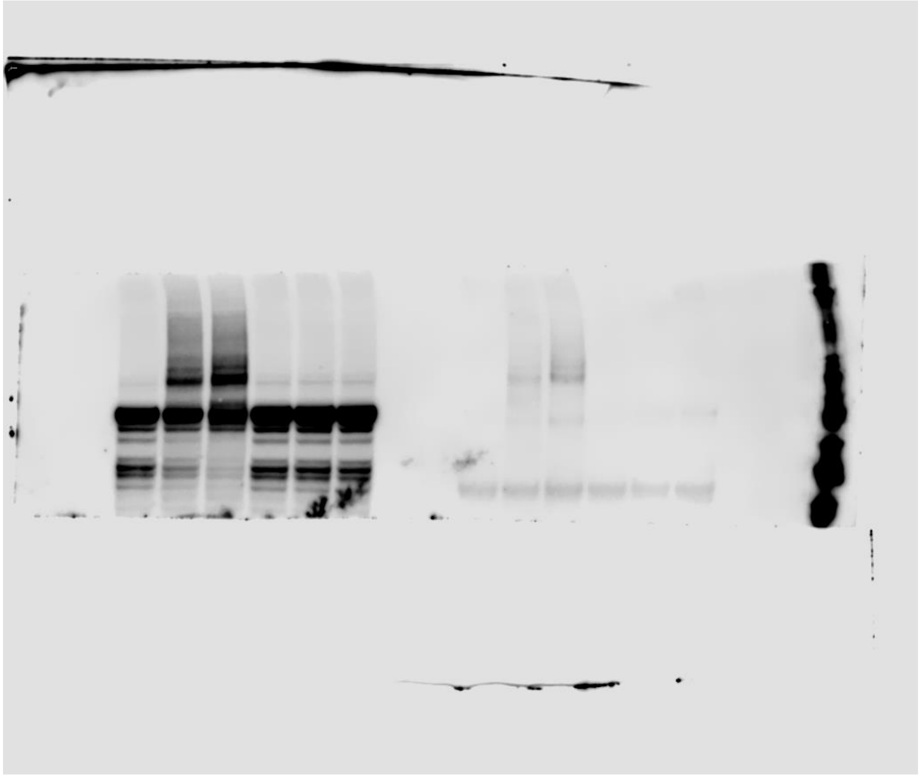

Figure 6F, right middle panel for IRAK1 in IPs

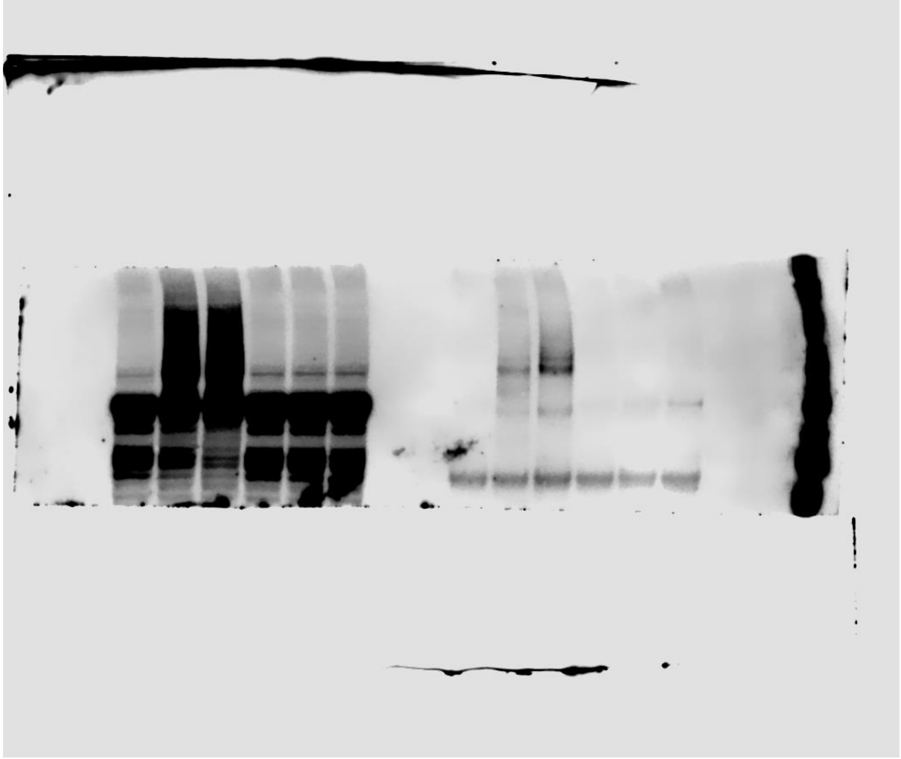

Figure 6F, bottom panel for IRAK4 in WCLs and IPs

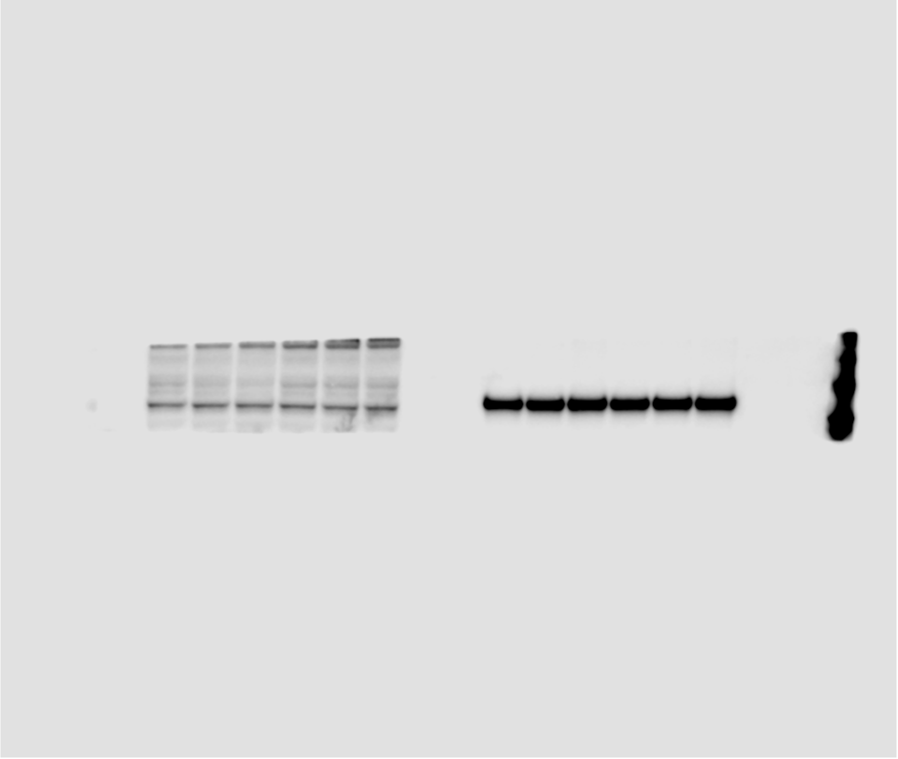

Supplement: Supplementary file 6 [file LSA-2023-02164_SdataF6.pdf]
