# Supplementary material for: Peptide derived from SLAMF1 prevents TLR4-mediated inflammation in vitro and in vivo
Source: Life Sci Alliance. 2023 Oct 3;6(12):e202302164. doi: 10.26508/lsa.202302164 (PMC10547912; doi:10.26508/lsa.202302164)

Source file for Figure 7

Peptide derived from SLAMF1 prevents TLR4-mediated inflammation *in vitro* and *in vivo*

Figure 7A, top panel for MyD88 in lysates

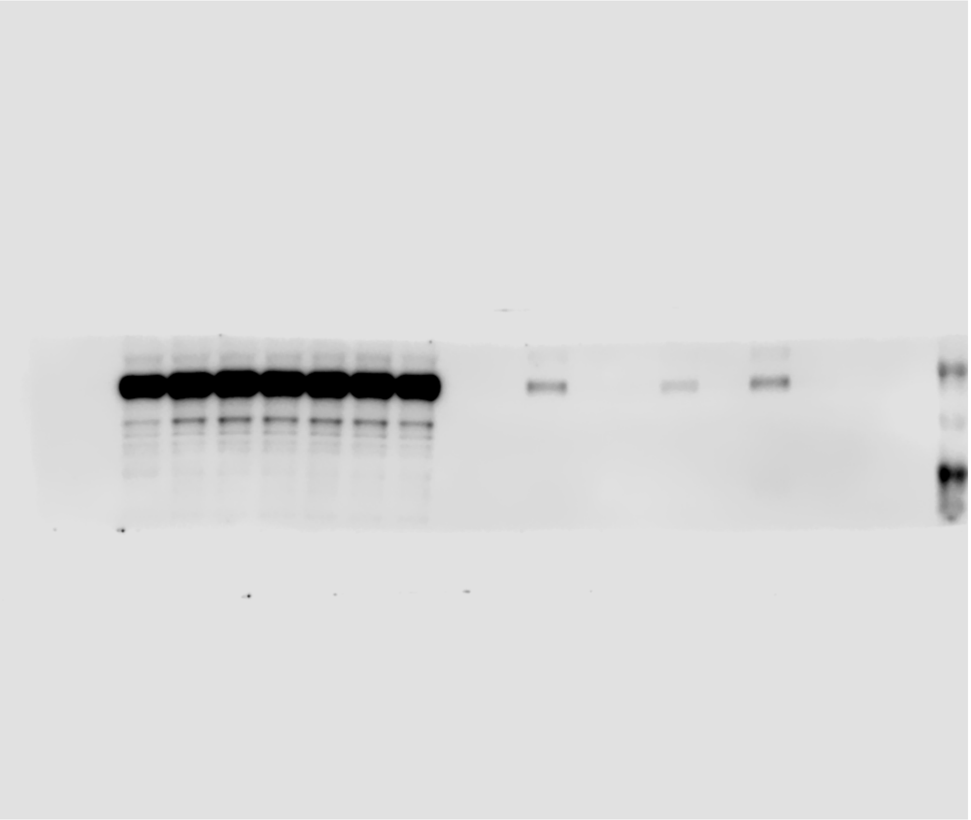

Figure 7A, top panel for MyD88 in lysates

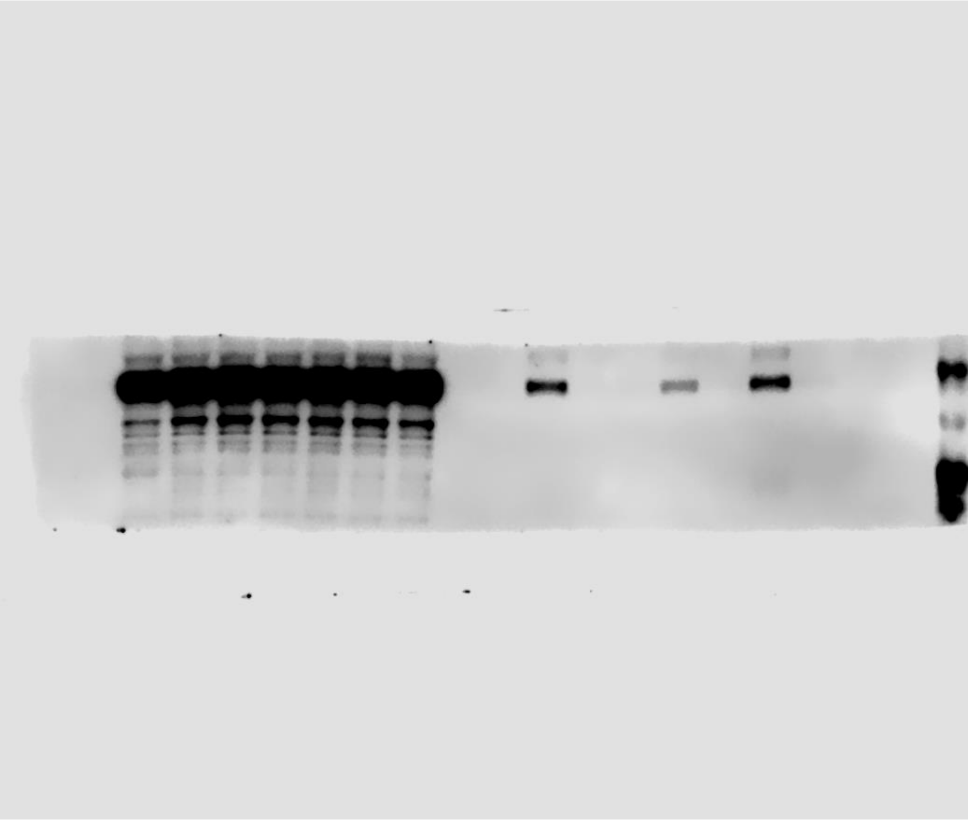

Figure 7A, bottom panel for TIRAP in lysates

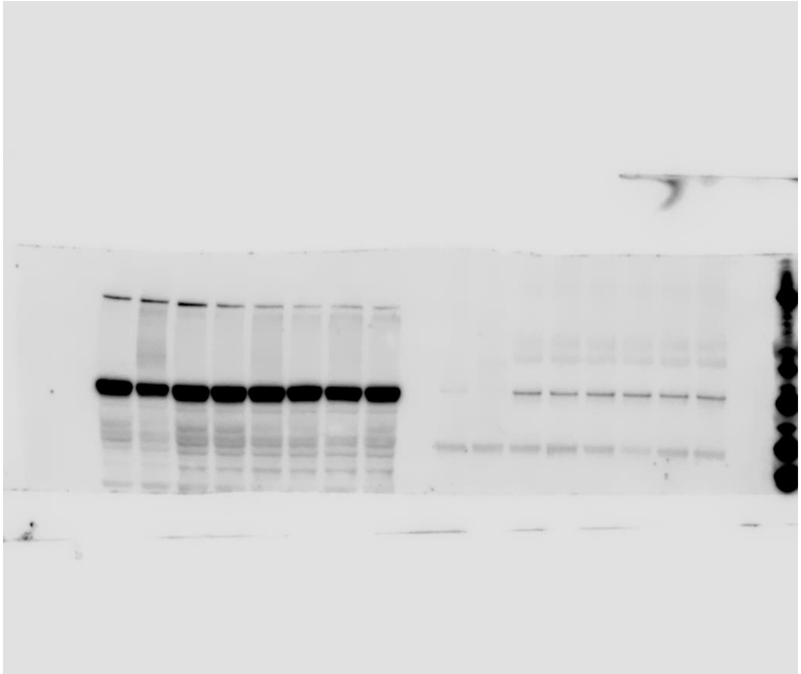

Figure 7A, bottom panel for TIRAP in IPs

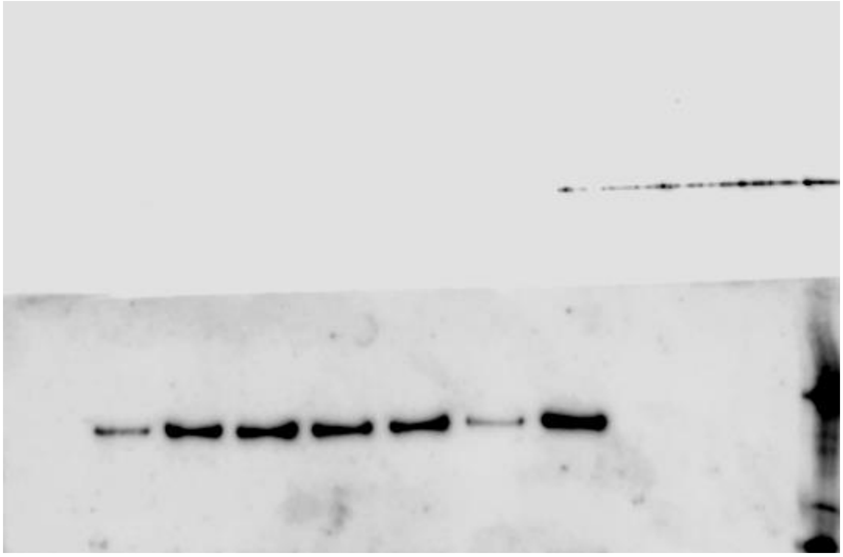

Figure 7B, top panel for MyD88 in lysates

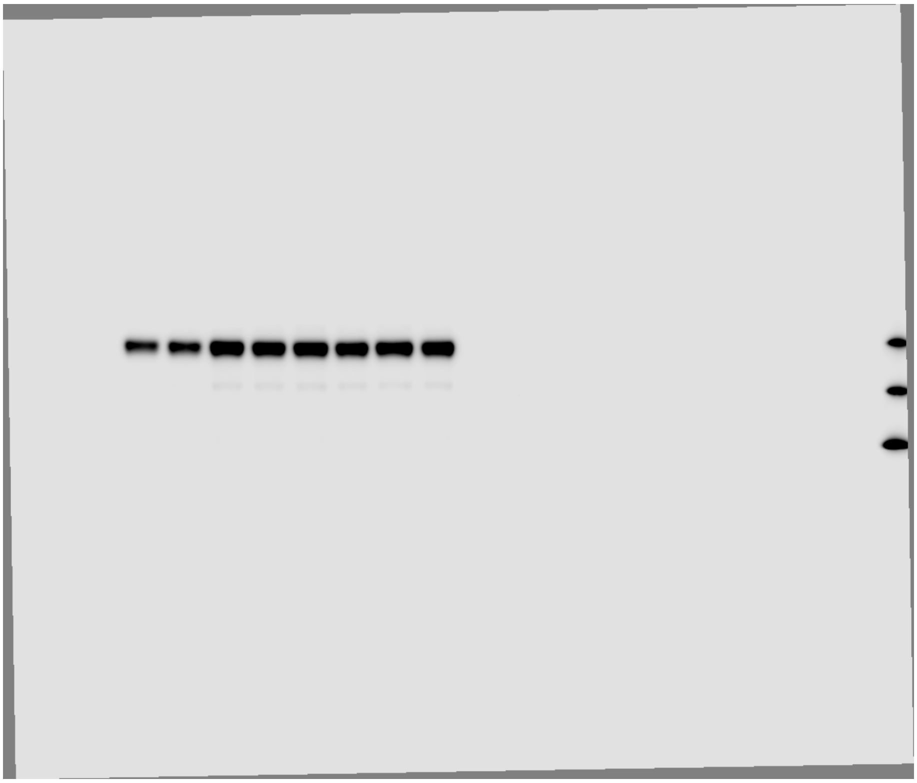

Figure 7B, top panel for MyD88 in IPs

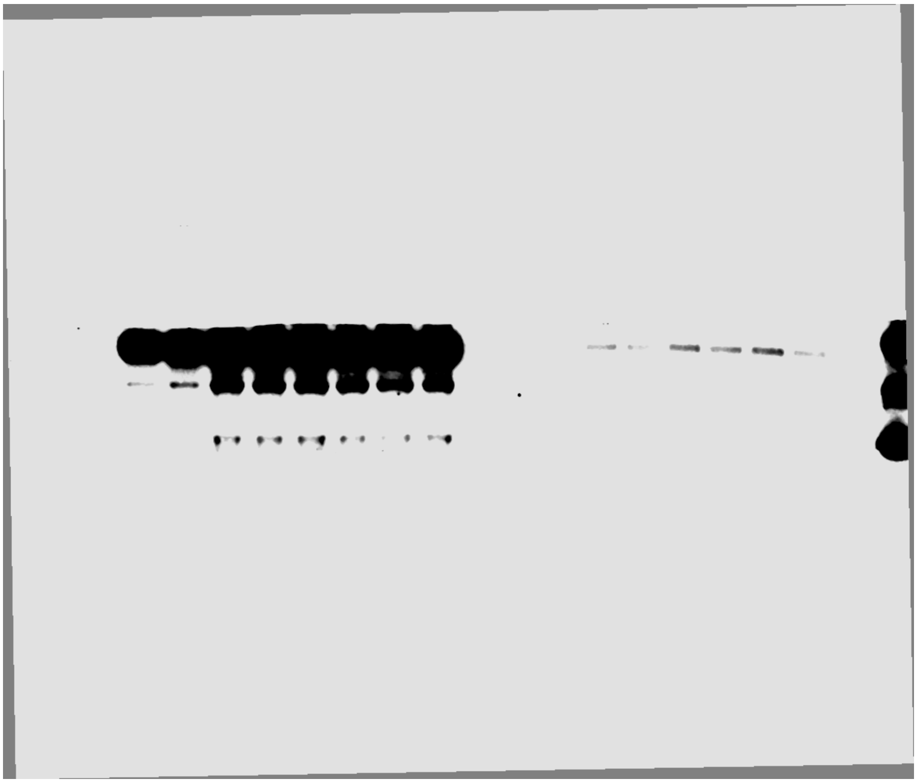

Figure 7B, middle panels for TIRAP in lysates

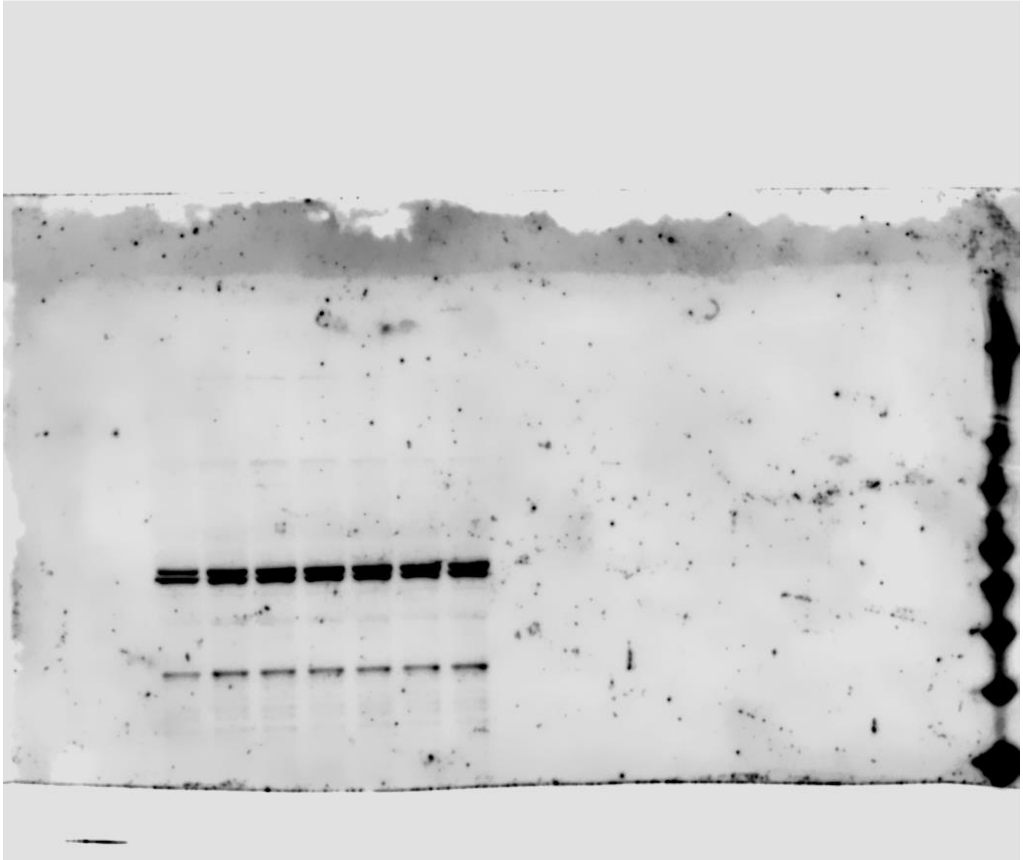

[illegible]

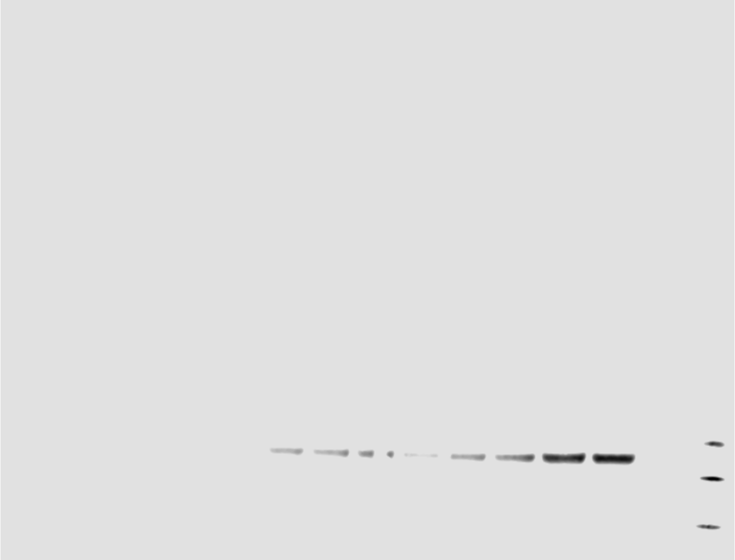

Figure 7B, bottom panel for TLR4^{FLAG} in lysates/input

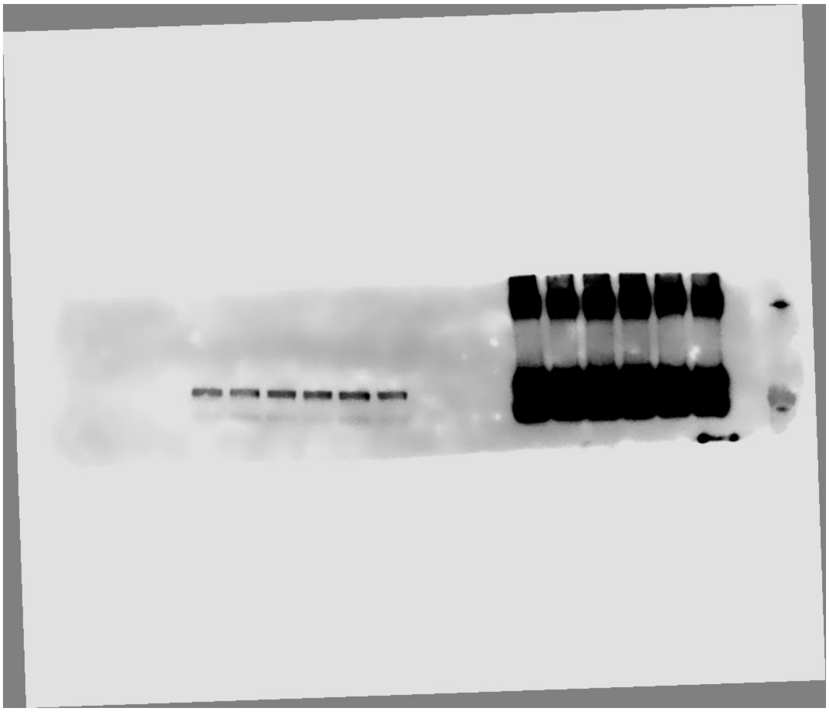

Figure 7B, bottom panel for TLR4^{FLAG} in IPs

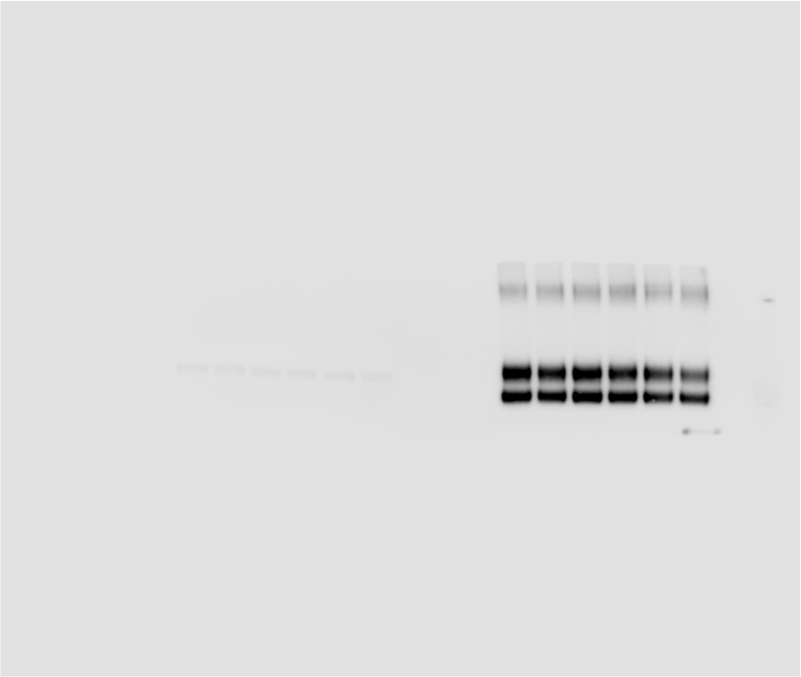

Figure 7C, top panel for IRAK1 in the lysates

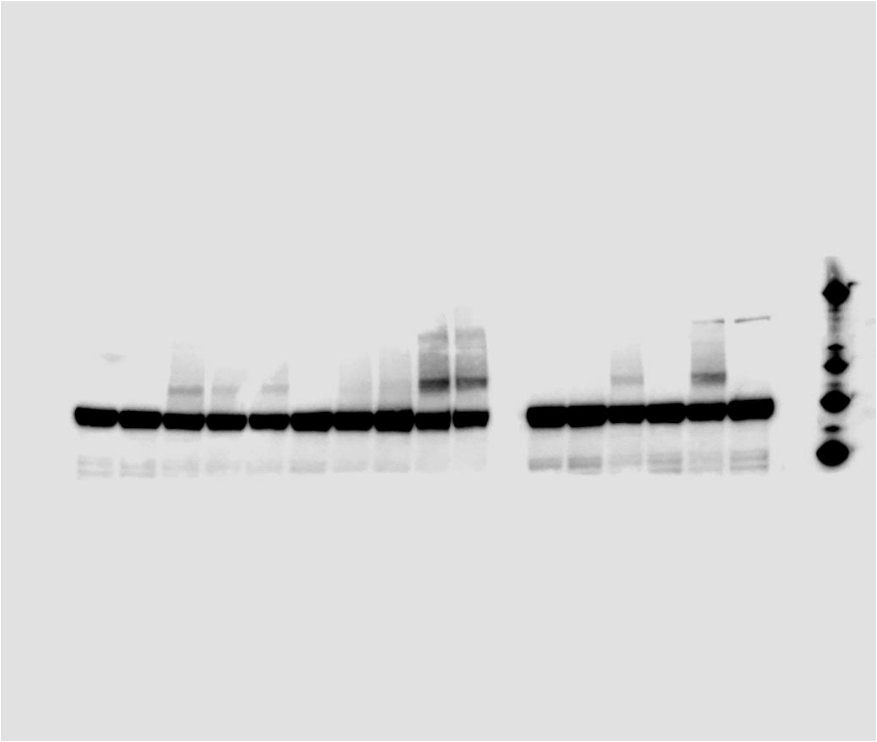

Figure 7C, top panel for IRAK1 in the IPs

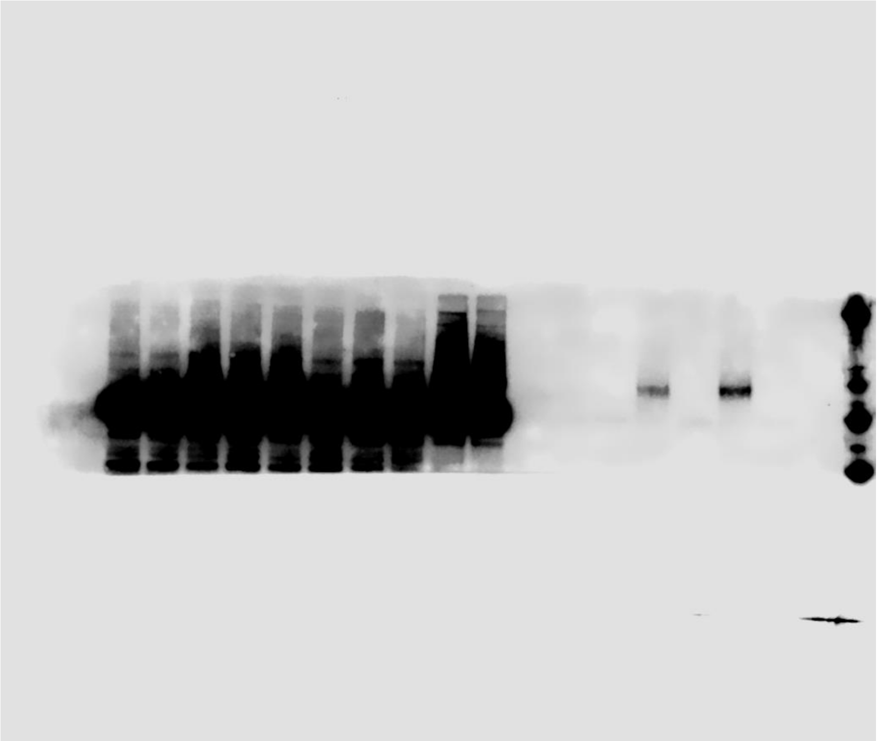

Figure 7C, middle panel for MyD88 in the lysates

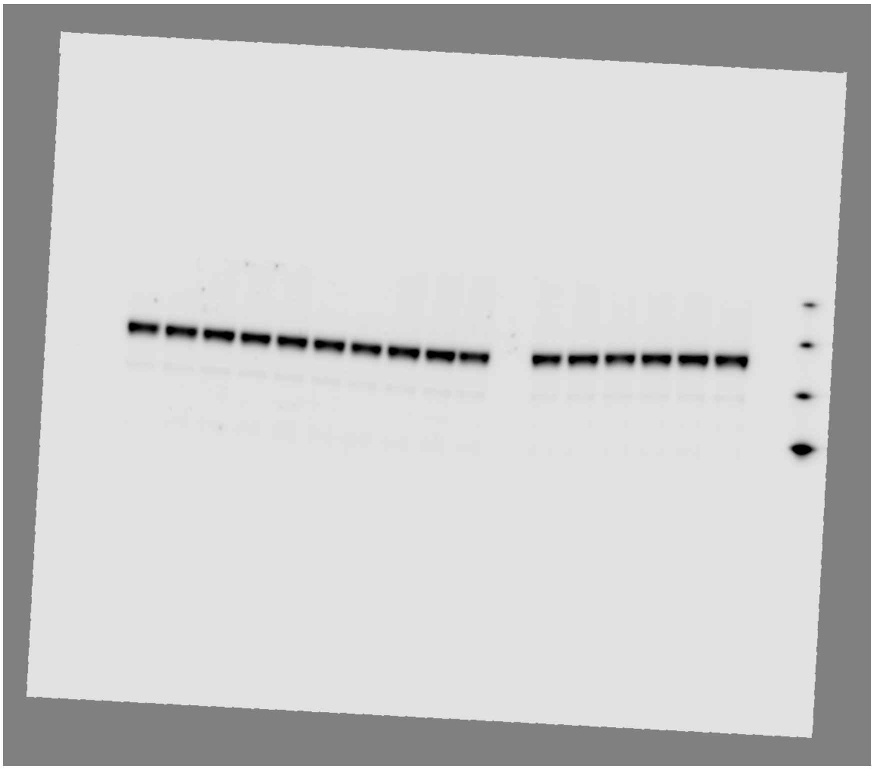

Figure 7C, middle panel for MyD88 in the IPs

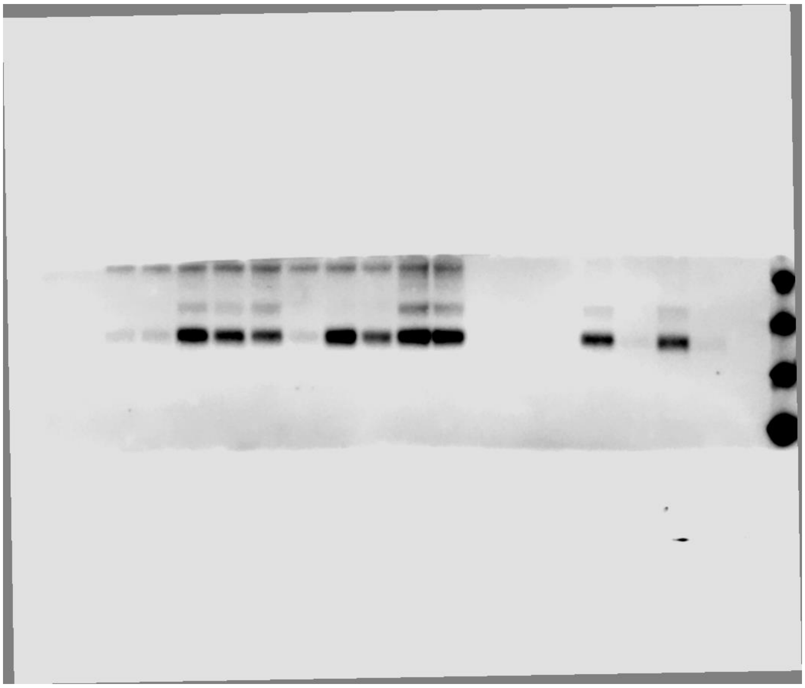

Figure 7C, bottom panel for TIRAP in the lysates

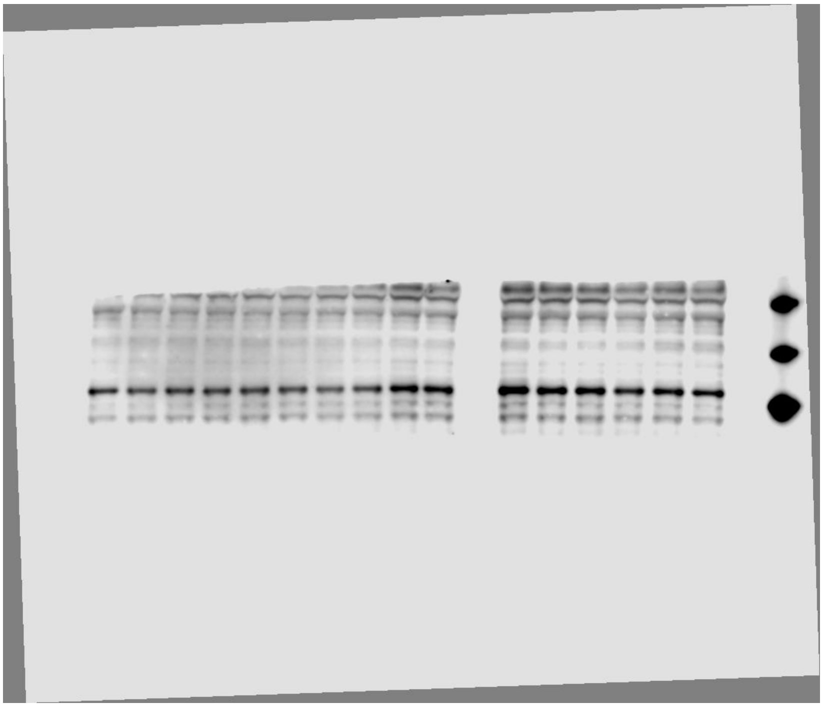

Figure 7C, bottom panel for TIRAP in the IPs

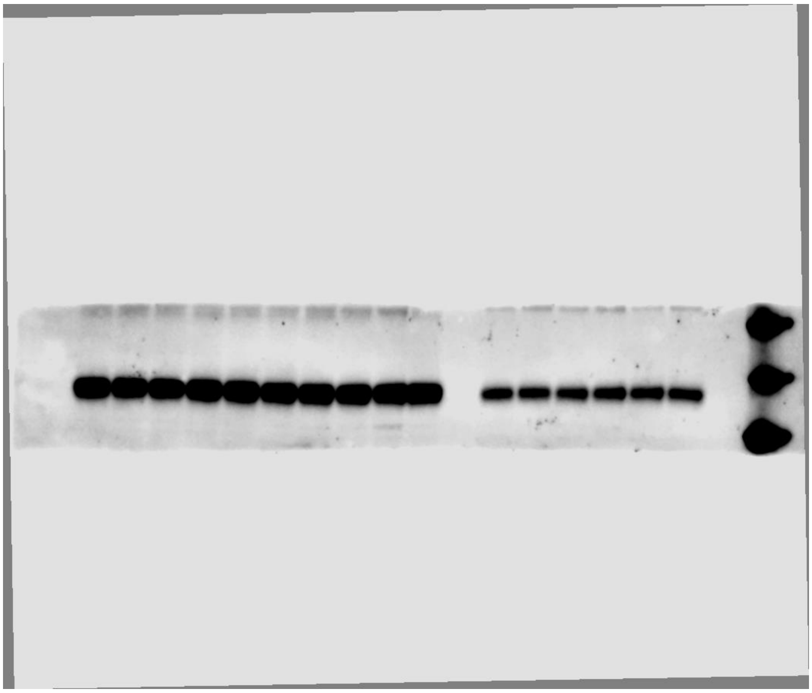

Figure 7D, top panel for MyD88 in the lysates

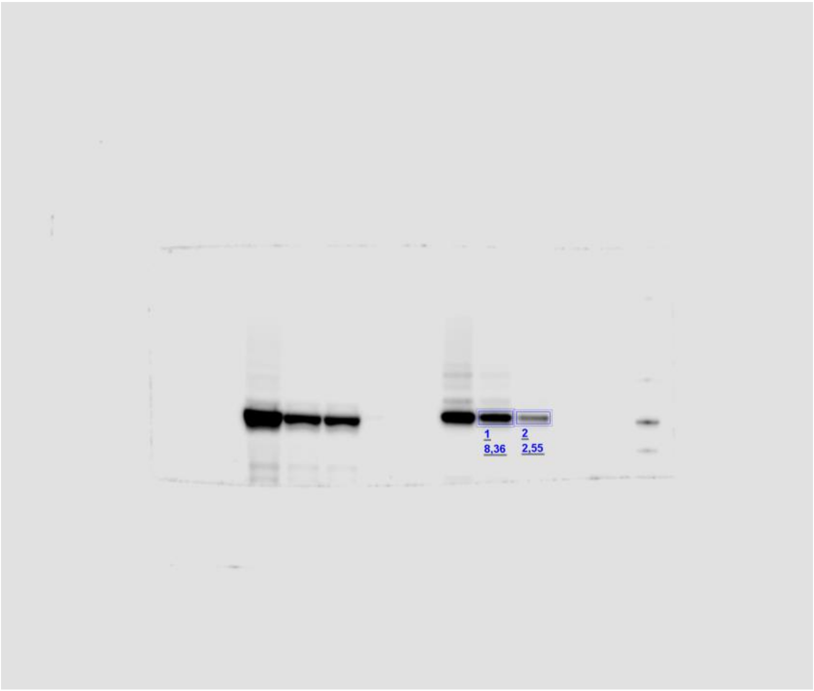

Figure 7D, top panel for MyD88 in the IPs

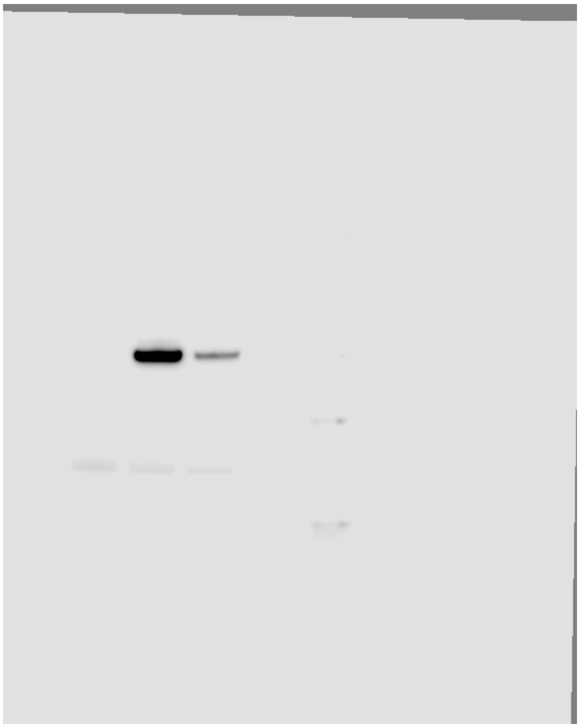

Figure 7D, bottom panel for TIRAP^{FLAG} in lysates and IPs

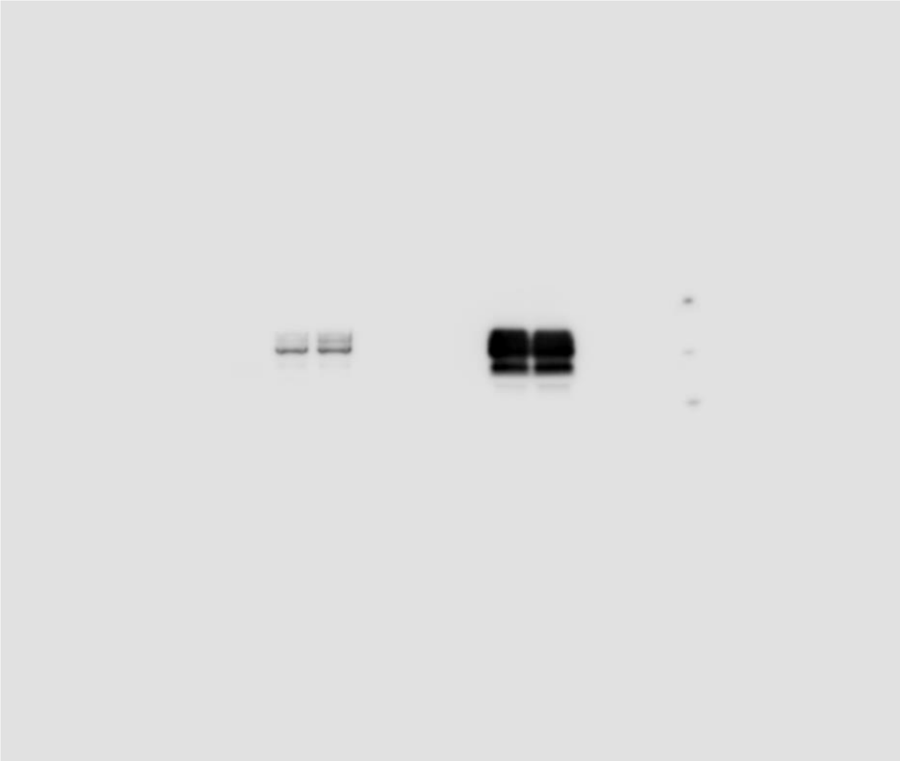

Figure 7E, top panel for TIRAP^{HA} in the lysates/input

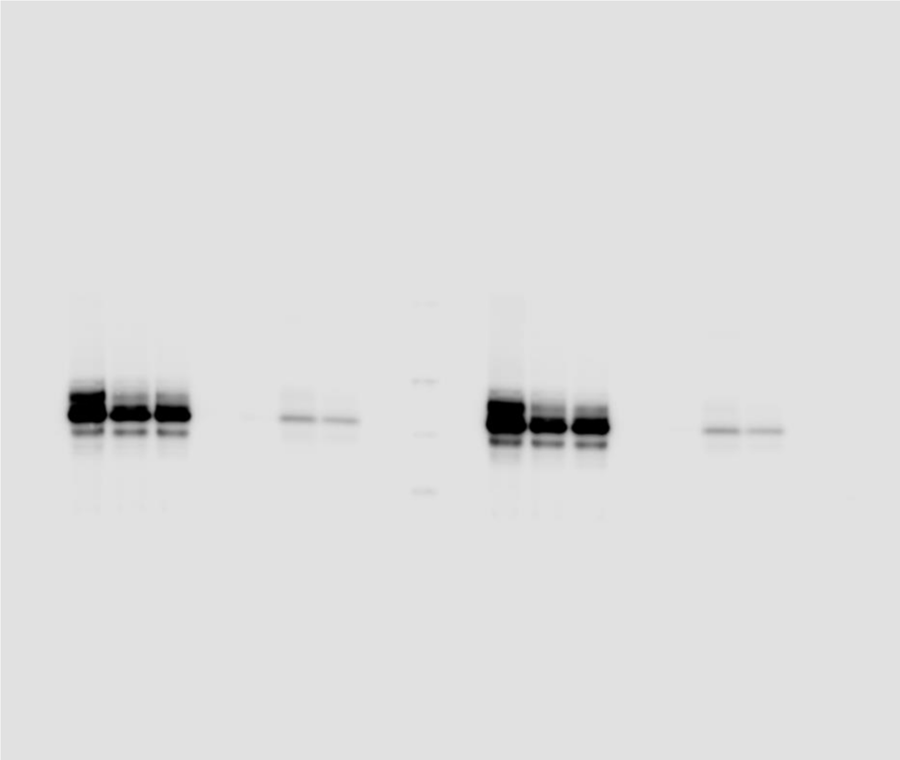

Figure 7E, top panel for TIRAP^{HA} in the lysates/input

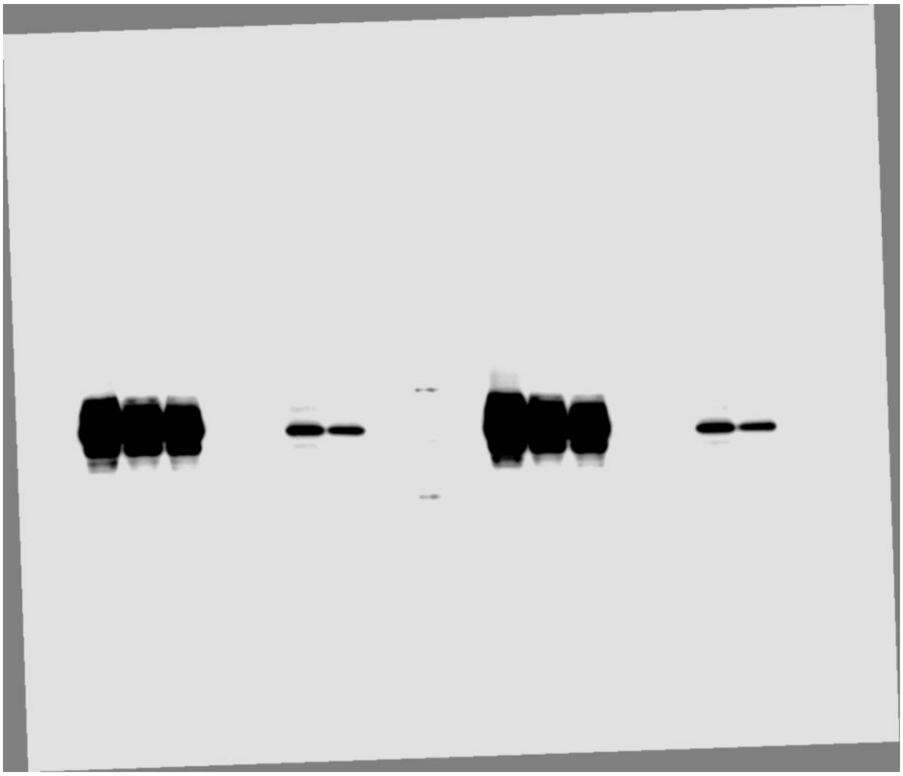

Figure 7E, bottom panel for TLR4^{FLAG} in the lysates

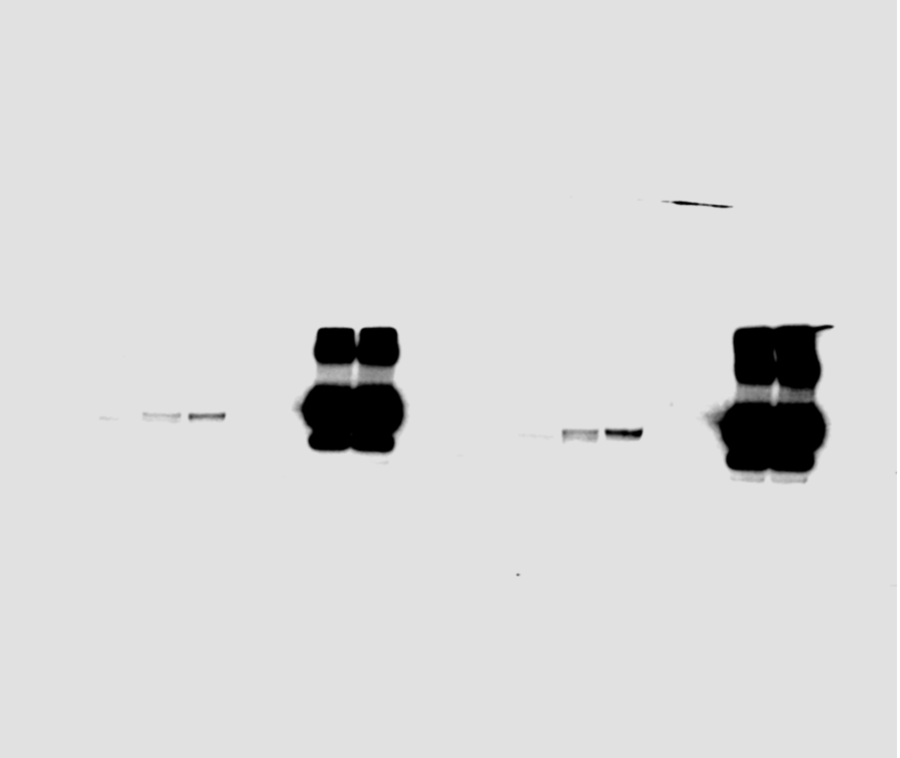

Figure 7E, bottom panel for TLR4^{FLAG} in the IPs

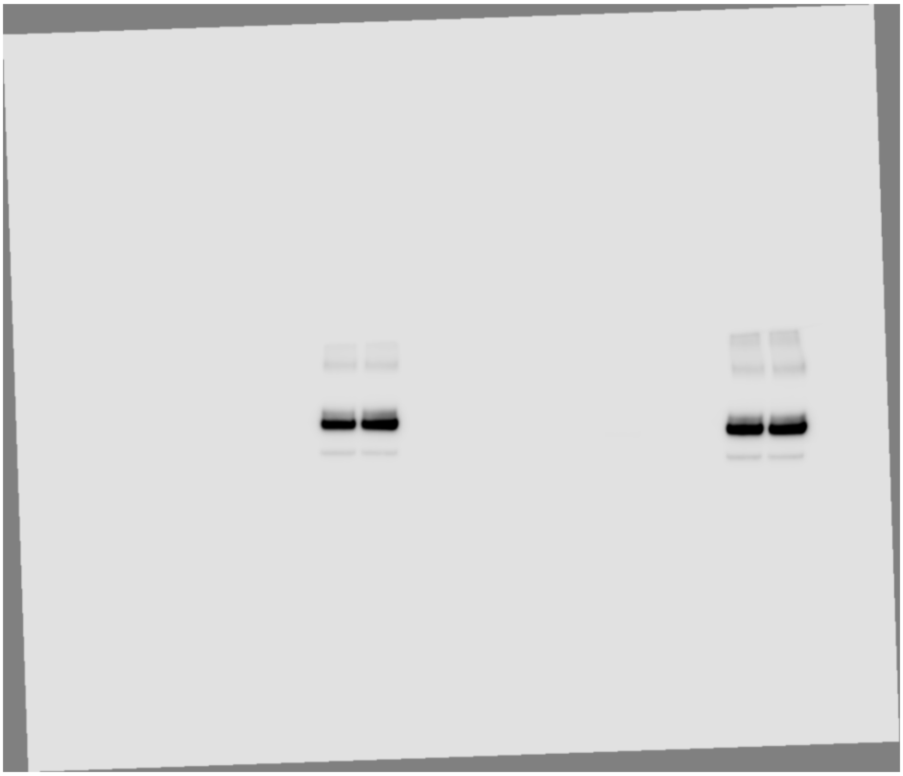

Supplement: Supplementary file 7 [file LSA-2023-02164_SdataF7.pdf]
