# Supplementary material for: Peptide derived from SLAMF1 prevents TLR4-mediated inflammation in vitro and in vivo
Source: Life Sci Alliance. 2023 Oct 3;6(12):e202302164. doi: 10.26508/lsa.202302164 (PMC10547912; doi:10.26508/lsa.202302164)

Source file for Figure 9

Peptide derived from SLAMF1 prevents TLR4-mediated inflammation *in vitro* and *in vivo*

Figure 9A, FLAG WB for WCLs

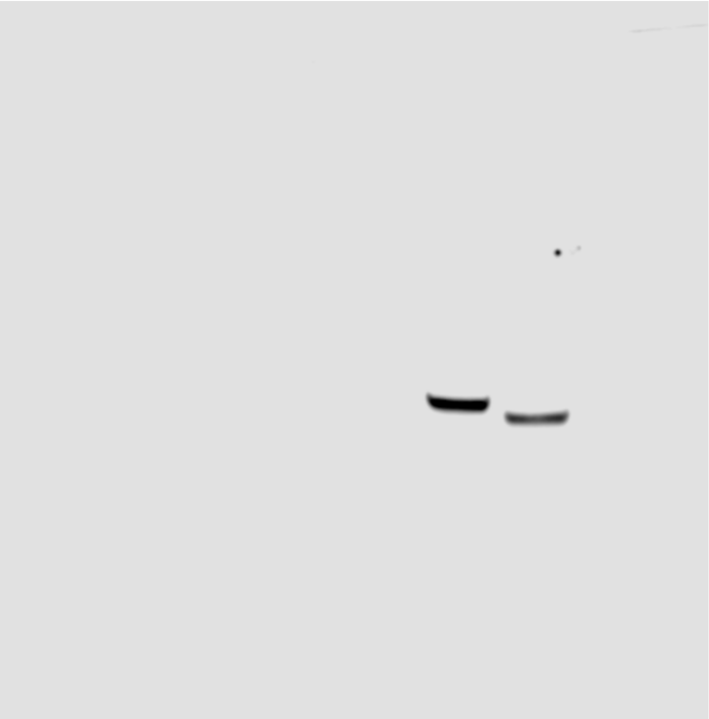

Figure 9A, FLAG WB for pull downs (PDs)

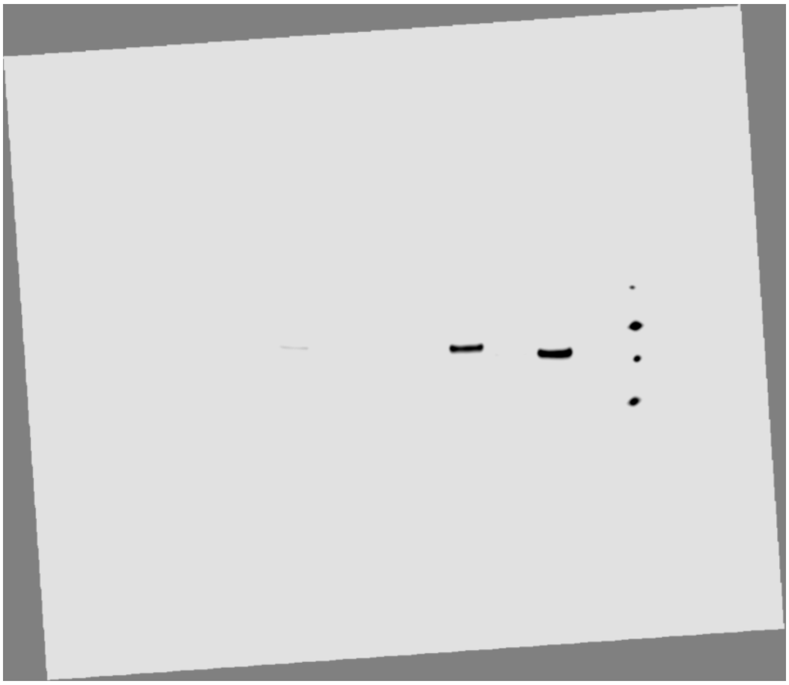

Figure 9B, IRAK1 panel

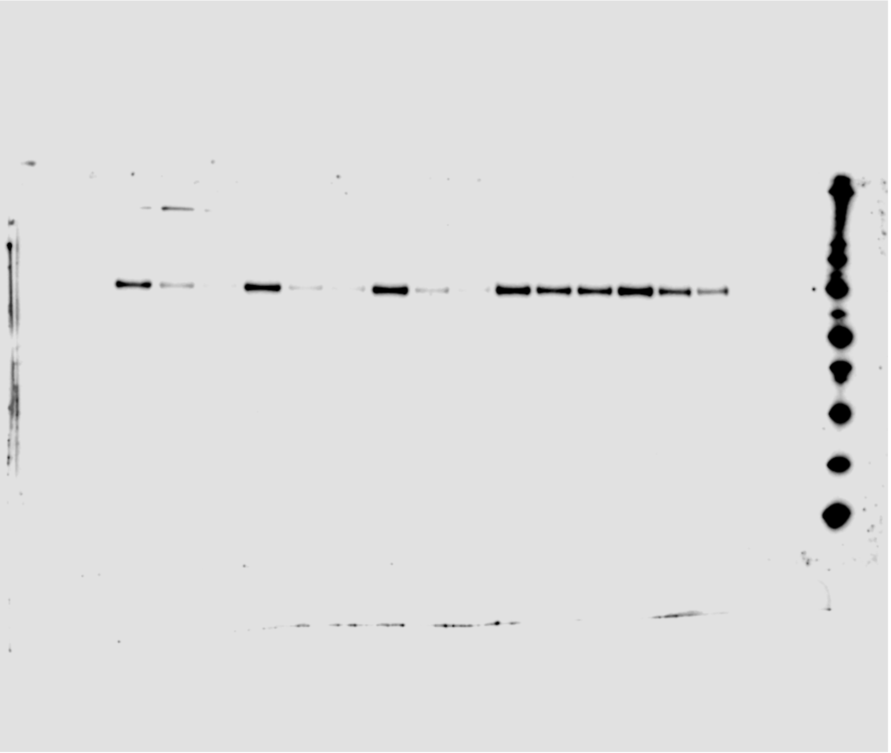

Figure 9B, pTAK1 panel

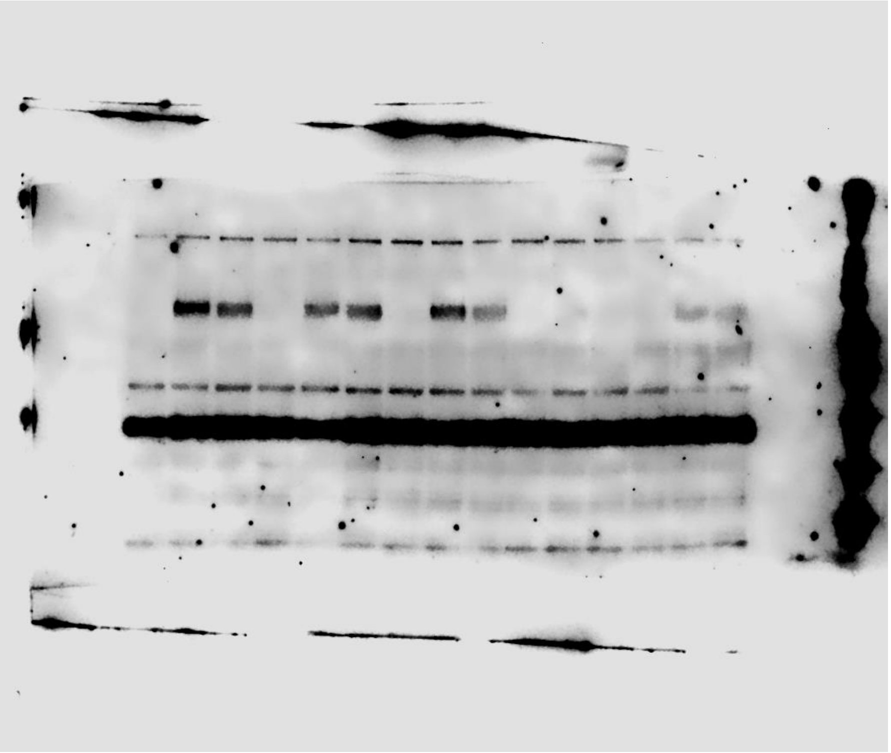

Figure 9B, pIκBα panel

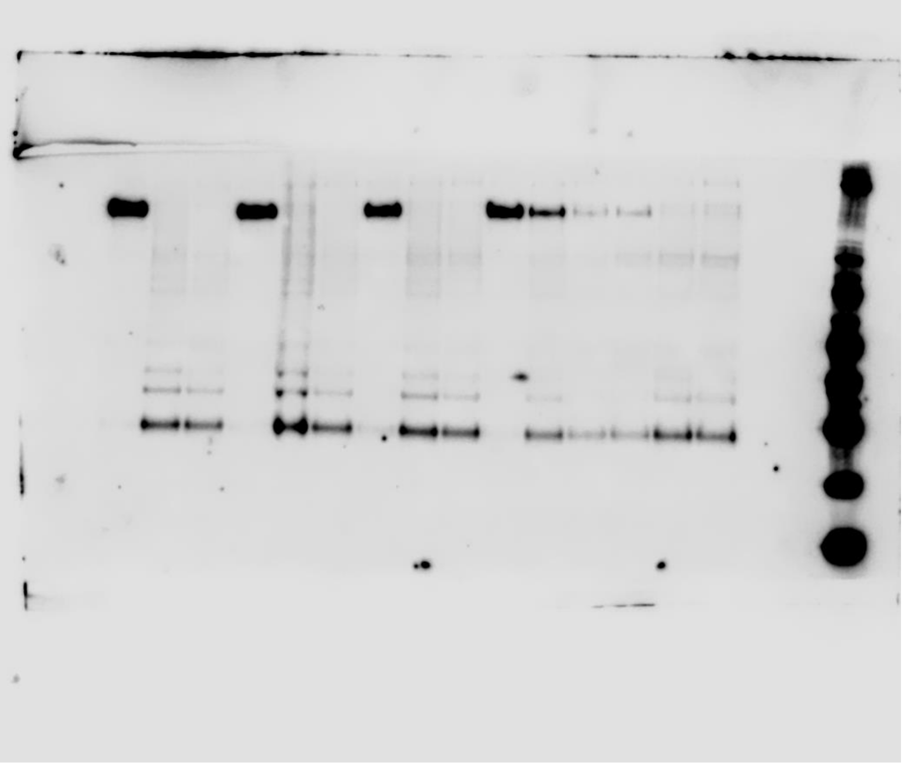

Figure 9B, p-p38 MAPK panel

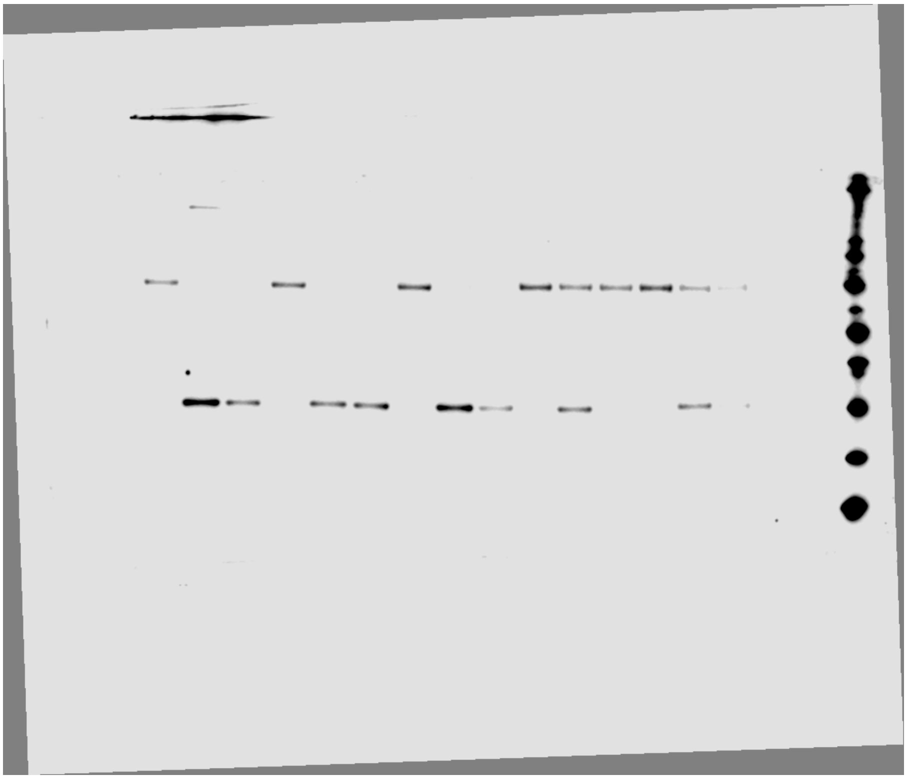

Figure 9B, beta-tubulin for the top group of WBs

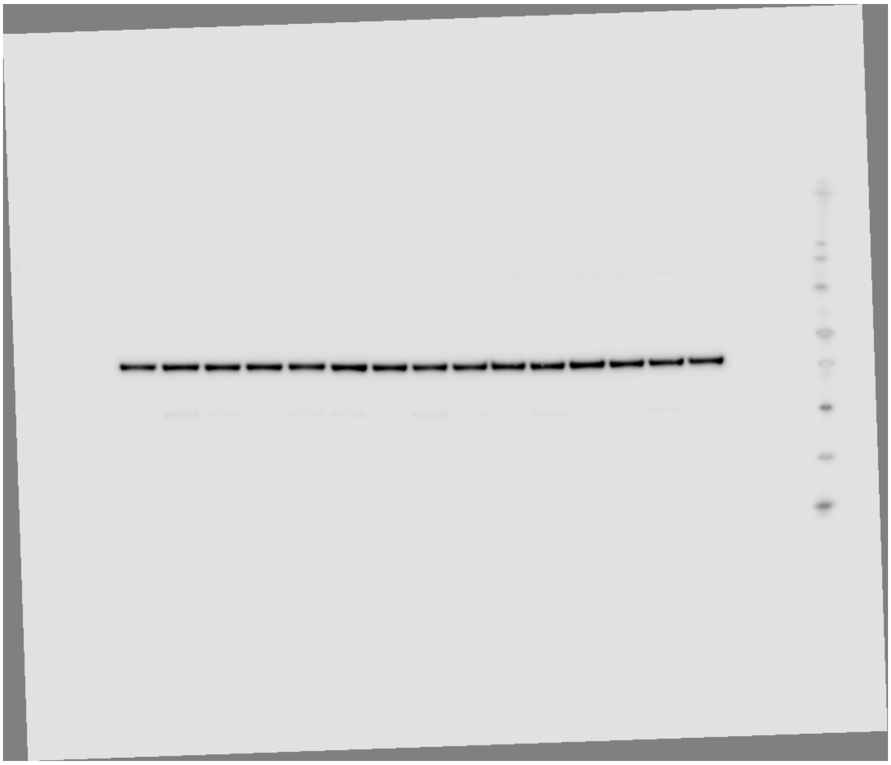

Figure 9B, pTBK1 panel

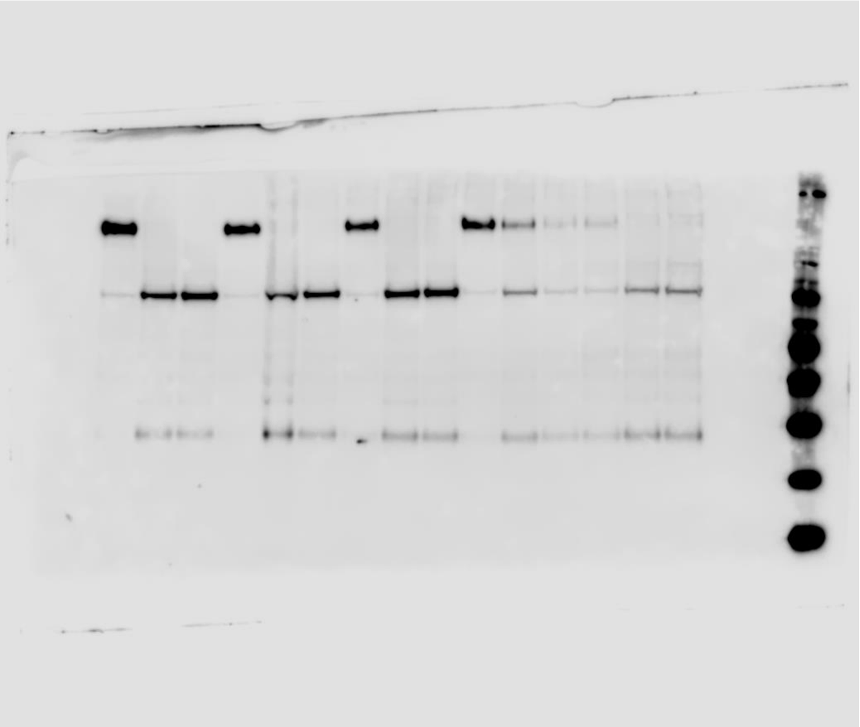

Figure 9B, beta-tubulin for pTBK1

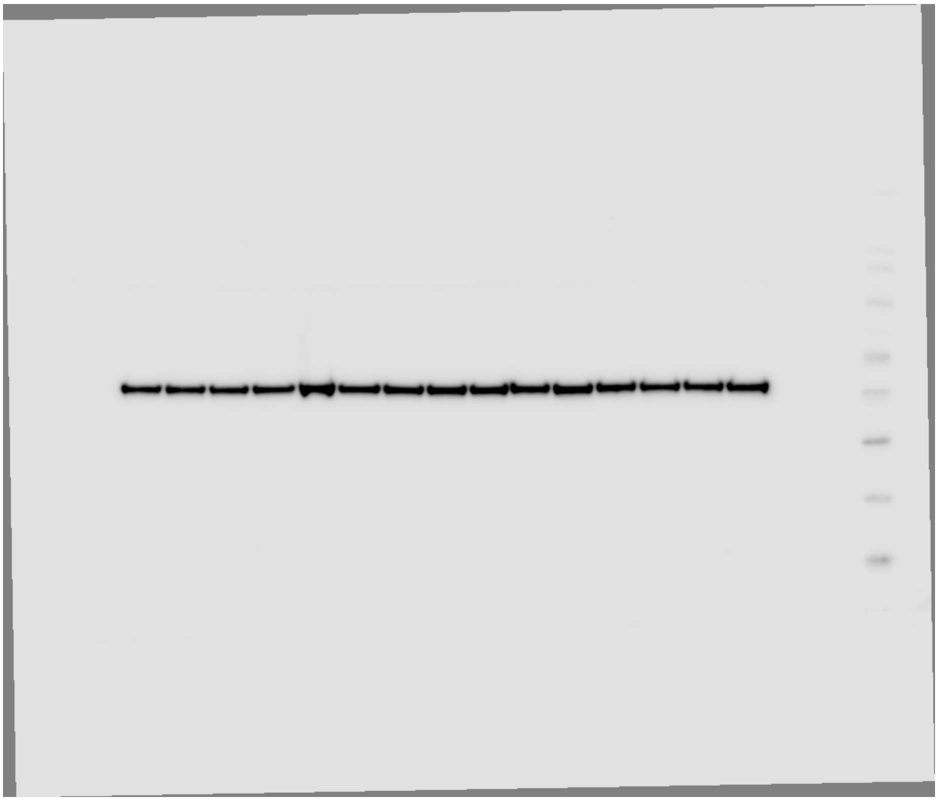

Supplement: Supplementary file 9 [file LSA-2023-02164_SdataF9.pdf]
