# Supplementary material for: Peptide derived from SLAMF1 prevents TLR4-mediated inflammation in vitro and in vivo
Source: Life Sci Alliance. 2023 Oct 3;6(12):e202302164. doi: 10.26508/lsa.202302164 (PMC10547912; doi:10.26508/lsa.202302164)
Supplement: Supplementary file 10 [file LSA-2023-02164_TableS2.docx]

**Table S2 - Results of the ELISA (for IFNβ) and BioPlex assays for the murine plasma samples.** Statistical significance evaluated by Mann-Whitney test and significant data marked by color code (description in notes to the table). Significance was evaluated according to the matching control water-treated animal group – “H2O pre” (pre-treatment) for P7 pre-treatment groups (2.5 nmol/g and 5 nmol/g) and – “H2O post” for P7 (5 nmol/g) post-treatment group.

| *treatment* | **Untreated** | | **H2O pre** | | **P7 (2.5 nmol/g) pre** | | **P7 (5 nmol/g) pre** | | **H2O post** | | **P7 (5 nmol/g) post** | |
| --- | --- | --- | --- | --- | --- | --- | --- | --- | --- | --- | --- | --- |
| *# animals* | *n = 12* | | *n = 11* | | *n = 11* | | *n = 11* | | *n = 12* | | *n = 11* | |
| *values* | mean | SEM | mean | SEM | mean | SEM | mean | SEM | mean | SEM | mean | SEM |
| **Mo Eotaxin** | 669.9 | 92.1 | 2450.2 | 422.8 | 1897.5 | 210.3 | 2670.5 | 207.6 | 2068.8 | 270.2 | 3848.5 | 401.7 |
| **Mo G-CSF** | 244.9 | 139.2 | 30555.7 | 3294.7 | 27545.1 | 3199.3 | 34923.9 | 3578.2 | 15160.6 | 1647.9 | 15860.0 | 2085.2 |
| **Mo GM-CSF** | 48.9 | 9.0 | 206.1 | 9.5 | 188.7 | 10.8 | 208.6 | 6.2 | 205.9 | 7.0 | 226.2 | 8.3 |
| **Mo IFN-g** | 6.1 | 0.8 | 43.9 | 3.2 | 36.9 | 2.4 | 46.0 | 3.4 | **50.1** | 2.6 | **61.2** | 3.3 |
| **Mo IL-1a** | 12.9 | 1.2 | 63.4 | 4.8 | 61.4 | 5.2 | 63.8 | 3.3 | 54.9 | 4.4 | 70.7 | 4.2 |
| **Mo IL-1b** | 2.2 | 0.3 | 21.0 | 1.8 | 17.5 | 1.7 | 17.4 | 1.1 | 17.0 | 0.9 | 17.5 | 1.3 |
| **Mo IL-2** | 21.5 | 11.0 | **66.8** | **3.2** | **56.8** | **3.8** | 83.6 | 16.8 | **68.1** | **3.4** | **112.9** | **27.3** |
| **Mo IL-3** | 0.9 | 0.3 | **16.0** | 1.3 | **11.9** | 1.1 | **13.6** | 1.1 | 15.5 | 0.9 | 15.1 | 1.2 |
| **Mo IL-4** | 1.8 | 0.3 | 12.3 | 0.6 | 10.5 | 0.7 | 11.4 | 0.8 | 14.1 | 0.6 | 14.9 | 0.8 |
| **Mo IL-5** | 7.6 | 1.3 | 22.0 | 1.3 | 32.5 | 5.4 | 36.2 | 4.3 | 26.2 | 1.4 | 25.9 | 1.4 |
| **Mo IL-6** | 13.7 | 4.2 | 12042.4 | 1300.6 | 10878.0 | 1093.0 | 15351.6 | 1251.3 | 9802.8 | 862.1 | 10088.4 | 914.0 |
| **Mo IL-9** | 13.7 | 7.1 | 67.0 | 2.9 | 60.7 | 3.4 | 67.6 | 1.9 | 75.6 | 3.0 | 86.8 | 3.1 |
| **Mo IL-10** | 29.3 | 4.1 | **1064.1** | 154.1 | **1340.4** | 216.9 | **2345.4** | 223.3 | **1693.9** | 196.3 | **2954.9** | 252.5 |
| **Mo IL-12(p40)** | 629.3 | 36.3 | **13471.6** | 1544.3 | **7271.4** | 916.3 | **6090.1** | 889.0 | **8929.7** | 984.7 | **5954.1** | 1120.3 |
| **Mo IL-12(p70)** | 2.6 | 1.7 | 159.1 | 15.2 | 139.8 | 13.6 | 183.3 | 14.2 | **197.2** | **15.5** | **264.2** | **17.9** |
| **Mo IL-13** | 31.9 | 6.7 | **157.4** | **8.6** | **133.7** | **8.1** | 158.4 | 14.5 | 149.4 | 8.3 | 160.1 | 8.9 |
| **Mo IL-17A** | 16.6 | 4.1 | **24.9** | **6.6** | 29.0 | 4.6 | **63.5** | **12.4** | 51.8 | 8.5 | 87.9 | 14.2 |
| **Mo KC** | 132.4 | 31.2 | 18247.7 | 1596.9 | 20062.1 | 2016.0 | 21562.8 | 989.5 | 20023.2 | 2197.6 | 24755.4 | 2424.4 |
| **Mo MCP-1** | 393.8 | 20.7 | 47550.0 | 4808.8 | 36607.7 | 4102.6 | 34576.5 | 2993.5 | 51075.5 | 5113.5 | 48320.2 | 5439.9 |
| **Mo MIP-1a** | 1.4 | 0.2 | 1853.7 | 275.8 | 1501.8 | 219.0 | 1495.0 | 168.5 | 1959.2 | 170.9 | 1788.4 | 180.2 |
| **Mo MIP-1b** | 50.8 | 6.7 | 20027.9 | 1673.9 | 18811.4 | 1518.9 | 19895.6 | 898.7 | 21104.1 | 1377.1 | 20541.7 | 1788.3 |
| **Mo RANTES** | 56.9 | 11.6 | **995.7** | **146.9** | **598.8** | **69.9** | 818.7 | 67.0 | 558.4 | 59.6 | 520.4 | 50.8 |
| **Mo TNF-a** | 14.3 | 2.6 | 1448.9 | 365.1 | 800.6 | 210.5 | 663.9 | 142.6 | 8005.3 | 1129.2 | 9678.6 | 2158.8 |
| **Mo IFN-beta** | **N.D.** |  | **712.5** | **397.5** | **251.8** | **203.3** | **354.8** | **155.6** | **531.7** | **171.3** | **270.7** | **39.6** |
|  |  |  |  |  |  |  |  |  |  |  |  |  |
|  | significant increase when compared to water (H2O) control | | | | | | | | | | | |
|  | significant decrease when compared to water (H2O) control | | | | | | |  |  |  |  |  |
| **N.D.** | not detected | |  |  |  |  |  |  |  |  |  |  |
